# Sex-, feeding-, and circadian time-dependency of P-glycoprotein expression and activity - implications for mechanistic pharmacokinetics modeling

**DOI:** 10.1038/s41598-019-46977-0

**Published:** 2019-07-19

**Authors:** Alper Okyar, Swati A. Kumar, Elisabeth Filipski, Enza Piccolo, Narin Ozturk, Helena Xandri-Monje, Zeliha Pala, Kristin Abraham, Ana Rita Gato de Jesus Gomes, Mehmet N. Orman, Xiao-Mei Li, Robert Dallmann, Francis Lévi, Annabelle Ballesta

**Affiliations:** 1 Department of Pharmacology, Faculty of Pharmacy, Istanbul University, Beyazit, Istanbul TR-34116 Turkey; 2 Division of Biomedical Sciences, Warwick Medical School, University of Warwick, Coventry, UK; 3 INSERM and Paris Sud university, UMRS 935, Team “Cancer Chronotherapy and Postoperative Liver Functions”, Campus CNRS, Villejuif, F-94807 France; 4 Università degli Studi G. d’Annunzio Chieti e Pescara, Institute for Advanced Biomedical Technologies, Chieti, Italy; 5 Department of Biostatistics and Medical Informatics, Faculty of Medicine, Ege University, Bornova, Turkey

**Keywords:** Molecular medicine, Chemotherapy, Drug delivery, Translational research, Differential equations

## Abstract

P-glycoprotein (*P-gp*) largely influences the pharmacokinetics (PK) and toxicities of xenobiotics in a patient-specific manner so that personalized drug scheduling may lead to significant patient’s benefit. This systems pharmacology study investigated *P-gp* activity in mice according to organ, sex, feeding status, and circadian time. Sex-specific circadian changes were found in *P-gp* ileum mRNA and protein levels, circadian amplitudes being larger in females as compared to males. Plasma, ileum and liver concentrations of talinolol, a pure P-gp substrate, significantly differed according to sex, feeding and circadian timing. A physiologically-based PK model was designed to recapitulate these datasets. Estimated mesors (rhythm-adjusted mean) of ileum and hepatic P-gp activity were higher in males as compared to females. Circadian amplitudes were consistently higher in females and circadian maxima varied by up to 10 h with respect to sex. Fasting increased *P-gp* activity mesor and dampened its rhythm. *Ex-vivo* bioluminescence recordings of ileum mucosae from transgenic mice revealed endogenous circadian rhythms of P-gp protein expression with a shorter period, larger amplitude, and phase delay in females as compared to males. Importantly, this study provided model structure and parameter estimates to refine PK models of any *P-gp* substrate to account for sex, feeding and circadian rhythms.

## Introduction

Permeability-glycoprotein (*P-gp*), also known as Multi-Drug Resistance 1 (MDR1) or ATP Binding Cassette B1 (ABCB1), is an important member of the ATP Binding Cassette (ABC) transporter family that plays a major role in pumping many endogenous substances and xenobiotics out of cells. In particular, *P-gp* function is critical for drug detoxification both in healthy and diseased tissues^1^. Differences in *P-gp* expression between and within subjects may be responsible for the large variabilities observed in drug accumulation in healthy tissues and associated drug toxicities. As drug tolerability remains a clinical issue, especially in oncology with estimated treatment-related mortality of up to 3%^2^, a better understanding of *P-gp* activity dynamics in specific organs of individual patients would help optimizing drug administration and reducing severe side effects. This may in turn circumvent pharmacologic resistance, a frequent cause of cancer treatment failures^3^.

According to extensive experimental and clinical investigations, both *P-gp* activity and the tolerability of *P-gp*-transported agents can largely vary as a function of sex, genetic polymorphisms, feeding patterns and circadian time^4–9^. Thus, optimizing *P-gp* substrates administration represents a multi-factorial challenge which, in our views, could be best addressed using systems pharmacology. Such inter-disciplinary approaches offer to develop quantitative frameworks mechanistically representing the drug pharmacokinetics-pharmacodynamics (PK-PD) at the molecular scale. It then integrates multi-type datasets to generate testable predictions in experimental models and in patients, following scaling up to fit human situations^10^.

Thus, experimental approaches alone have not been able to draw any clear conclusion regarding a possible sex-dependence in *P-gp* expression^5,6,11^. Positive studies found an increased *P-gp* expression in the liver of men compared to women^6,12^. Sex-related variations were also reported for both tolerability and therapeutic response of anticancer drugs in laboratory animals and in cancer patients^6,9^. Worse toxicities and better response rates and survival have been consistently reported for women as compared to men on cancer chemotherapy for the vast majority of cancer types^13,14^. This may be explained in part by increased drug accumulation resulting from low *P-gp* expression in tissues of females as compared to males. Of note, none of these studies specified the circadian time of *P-gp* determination or the feeding patterns.

*P-gp*, like many transporters, is regulated by nuclear receptors, such as C-androstane receptors, that are in turn regulated by circadian clock-controlled transcription factors^15^. The mammalian circadian timing system (CTS) involves a hierarchical network of molecular clocks that reside within each cell and are coordinated by the suprachiasmatic nuclei (SCN), a hypothalamic pacemaker, that can be entrained to exactly 24 h by external cues. The CTS rhythmically regulates most cellular functions involved in drug absorption, distribution, metabolism, elimination and toxicity (ADMET). Interestingly, the mRNA expression of *Abcb1* which encodes for the main constituent of *P-gp*, was rhythmic in synchronized cultures of human colorectal cancer Caco-2 cells at confluence. Turning off the clock by silencing the core clock gene *BMAL1* ablated this circadian oscillation^16^. The expression of Abcb1a/b, the two rodent homologues of the human Abcb1 was also rhythmic in the liver and intestine of male rats and mice^17–19^. Further, the circadian variations of *Abcb1a/b* mRNA expressions were suppressed in the ileum of mice harboring a constitutive mutation of *Clock*^20^. P-gp activity also displayed a circadian pattern in the intestine of male rats and male monkeys^21,22^.

The circadian regulation of *P-gp* could indeed represent an important chronopharmacology mechanism to be considered specifically in order to optimize the delivery of medications through so-called chronotherapy schedules. Thus, the tolerability of anticancer *P-gp* substrates can vary by up to several-fold according to their time of administration within the 24-h span^23^. For instance, single agent docetaxel or doxorubicin, two *P-gp* substrates, were significantly best tolerated by mice following dosing near the middle of the rest span and most toxic 12 h later, during the nocturnal activity phase^24^. The cellular PK-PD of irinotecan, an anticancer agent whose efflux is mediated by *P-gp* among other ABC transporters, varied significantly according to circadian timing in clock-proficient but not in clock-deficient Caco-2 cells^16^. Furthermore, the dosing time dependency in PK (chronoPK) and toxicity (chronotoxicity) of irinotecan significantly varied according to sex and genetic background in mice^25–27^. Similarly, large sex-related differences were found in mice regarding the chronotoxicity of theprubicin, another *P-gp* substrate anticancer drug^28–30^. Randomized clinical trials and meta-analyses in cancer patients further suggested that the circadian timing associated with least toxicity and/or efficacy of cancer chemotherapy could largely differ between men and women^9,23^.

Feeding patterns also seemed to influence ABC transporter expressions and activities, thus altering the therapeutic index of many xenobiotics, including anticancer agents^31^. Indeed, 12 to 24 h fasting increased the hepatic expression of *P-gp* and other ABC transporters through *Pparα* pathway activation in male mice^8^, or through *Nrf2* up-regulation both in male mice and in Huh-7 human male liver cancer cell line^7^. Hence, proper feeding patterns might up-regulate ABC transporters in healthy tissues, thus reducing chemotherapy side effects^31^. In addition, feeding status influenced the chronobiology of the gastro-intestinal tract of mammals, and in particular the circadian expression of hepatic and intestinal transporters^32–35^. Thus, restricted feeding for a few hours over several consecutive days phase shifted the intestinal transport rhythms in such a way that transporter function increased before the programmed feeding^36^. Similarly, feeding restricted to the light phase shifted the rhythm of *Abcb1a* mRNA expression in the male rat intestine^37^.

Here, we aimed to provide a physiologically-based modeling framework encompassing sex, feeding patterns and circadian rhythms, for the quantitative study of the pharmacology of *P-gp* drug substrates, towards the optimization of their dosing time and schedule.

## Results

### Sex-specific circadian changes in *P-gp* expression in ileum

**Figure 1:**
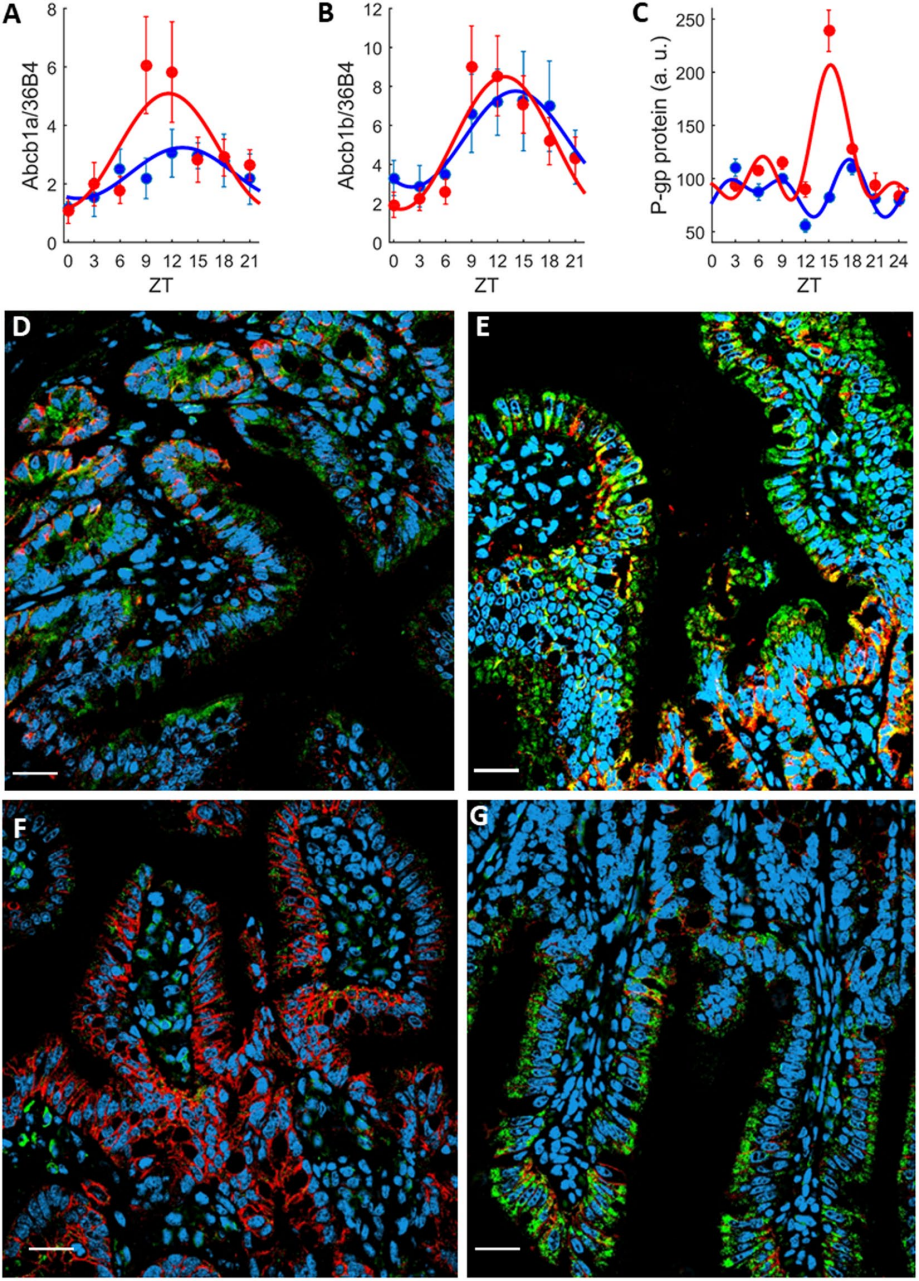
Sex-specific *P-gp* expression in the Ileum mucosa. (**A**–**C**) Circadian patterns of *Abcb1a* (**A**) *and Abcb1b* (**B**) mRNA expression, and whole *P-gp* protein (**C**) in female (red) and male (blue) *ad libitum* fed mice. Each data point is the average (±SEM) of 5 animals. Solid lines are the best-fit cosinor (Table S1). Regarding mRNAs, a 24 h-rhythm was found by the cosinor analysis for Abcb1a (male, p = 0.042; female, p = 0.0017), and Abcb1b (male, p = 0.02; female, p < 0.001). For P-gp protein, a 24 h-rhythm with significant 12 h- and 8 h-harmonics (p < 0.001 for all harmonics) was found in females and a 12 h-rhythm with a 8 h-harmonic was found in males (p = 0.002 and p = 0.001, respectively). (**D**–**G**) Confocal immunohistochemistry imaging of ileum *P-gp* protein expression at ZTs of minimum and maximum expression that were in female mice at ZT3 (**D**) and ZT15 (**E**), and in male mice at ZT12 (**F**) and ZT18 (**G**). Pictures represent merged images showing cell nuclei (blue), *P-gp* (green), *β-catenin* (red) and co-localization of P-gp and *β-catenin* (yellow). The scale bar represents 20 µm.

Statistically significant circadian rhythms were found for both *Abcb1a* and *Abcb1b* mRNA expressions in the ileum mucosa, yet with differences according to sex (ZT effect from ANOVA on both sexes, p = 0.002 for *Abcb1a*; p < 0.0001 for *Abcb1b*; Fig. 1A,B). Highest expressions of both mRNA levels were found from ZT9 to ZT12 in female mice, i.e. near the end of the light span, and from ZT12 to ZT18 in male mice, i.e. during the beginning of the dark span. ANOVA test of sex effect was not statistically significant either for *Abcb1a* or for *Abcb1b*. However, according to cosinor analysis, the 24-h mean of *Abcb1a* expression was higher in females as compared to males whereas those of *Abcb1b* were similar in both sexes (Table S1). A phase advance of approximately 1.5 h was found for both mRNA rhythms in females compared to males. The relative amplitudes of *Abcb1a* was 62% of the mesor in females as compared to 37% in males. Similar results were found for the relative amplitude of *Abcb1b*: 67% and 47% respectively.

Regarding *P-gp* protein expression, a 24 h-rhythm with a maximum at ZT15 was found for female mice whereas male mice displayed damped circadian variations. ANOVA identified statistically significant effects of ZT, sex and ZT*sex interactions (p < 0.0001 for all). A 24 h-rhythm and significant 12 h and 8 h harmonics were found with cosinor analysis in females whereas males displayed a rhythm of period 12 h with an 8 h-harmonic (Fig. 1C). Similarly, to what was found for the mRNA levels, i) the 24 h-means and amplitudes were higher in females as compared to males, ii) a phase advance of approximately 2 h was observed in the dominant rhythm of females as compared to males. *P-gp* protein expression was mainly observed in the plasma membrane of mucosa cells, as indicated by its co-localization with β-catenin, a reference plasma membrane marker (Fig. 1D,E, Figs S1, S2 in SI). β-catenin was used as a co-localization marker, and its changes in staining intensities according to sex and/or timing were not examined.

### Sex-specific circadian changes in *P-gp* expression in colon

**Figure 2:**
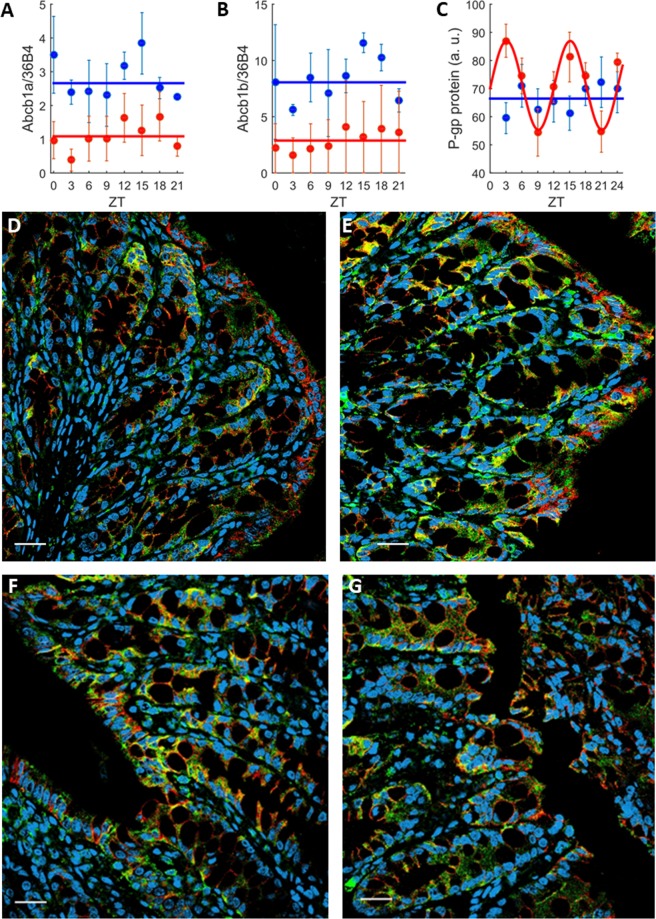
Sex-specific *P-gp* expression in the colon mucosa. (**A**–**C**) Circadian patterns of *Abcb1a* (**A**) and *Abcb1b* (**B**) mRNA expression, and whole *P-gp* protein (**C**). Each data point is the average (±SEM) of 5 animals. Solid lines are the best-fit cosinor (Table S2). Regarding mRNAs, no rhythms were demonstrated neither for males nor for females (all p values > 0.05). For P-gp protein, no rhythm was found for males (p > 0.05) and a 12 h-rhythm was found in females (p < 0.001). (**D**–**G**) Confocal immunohistochemistry imaging of colon *P-gp* protein expression at ZTs of minimum and maximum values that were in female mice at ZT9 (**D**) and ZT15 (**E**), and in male mice at ZT9 (**F**) and ZT15 (**G**). Pictures represent merged images showing cell nuclei (blue), *P-gp* (green), *β-catenin* (red) and co-localization of P-gp and *β-catenin* (yellow). The scale bar represents 20 µm.

The 24-h means of *Abcb1a* and *Abcb1b* mRNA expressions were significantly higher in males as compared to females (ANOVA testing sex effect, p < 0.0001), yet they were arrhythmic in both sexes (non-significant ZT effect with ANOVA and non-significant 24 h or 12 h rhythm detection with cosinor; Fig. 2A,B, Table S2 in SI). Regarding P-gp protein, 24 h-mean values were similar in males and in females (non-significant sex effect from ANOVA). No rhythm of P-gp protein expression in the colon was found for males (non-significant ZT effect from ANOVA and no rhythm with cosinor; Fig. 2C–G). However, a significant 12 h-rhythm with a relative amplitude of 22% of the mesor was found in females. The sex*ZT interaction was statistically significant (ANOVA, p = 0.04).

### Talinolol PK analysis according to sex, feeding conditions and circadian timing

**Figure 3:**
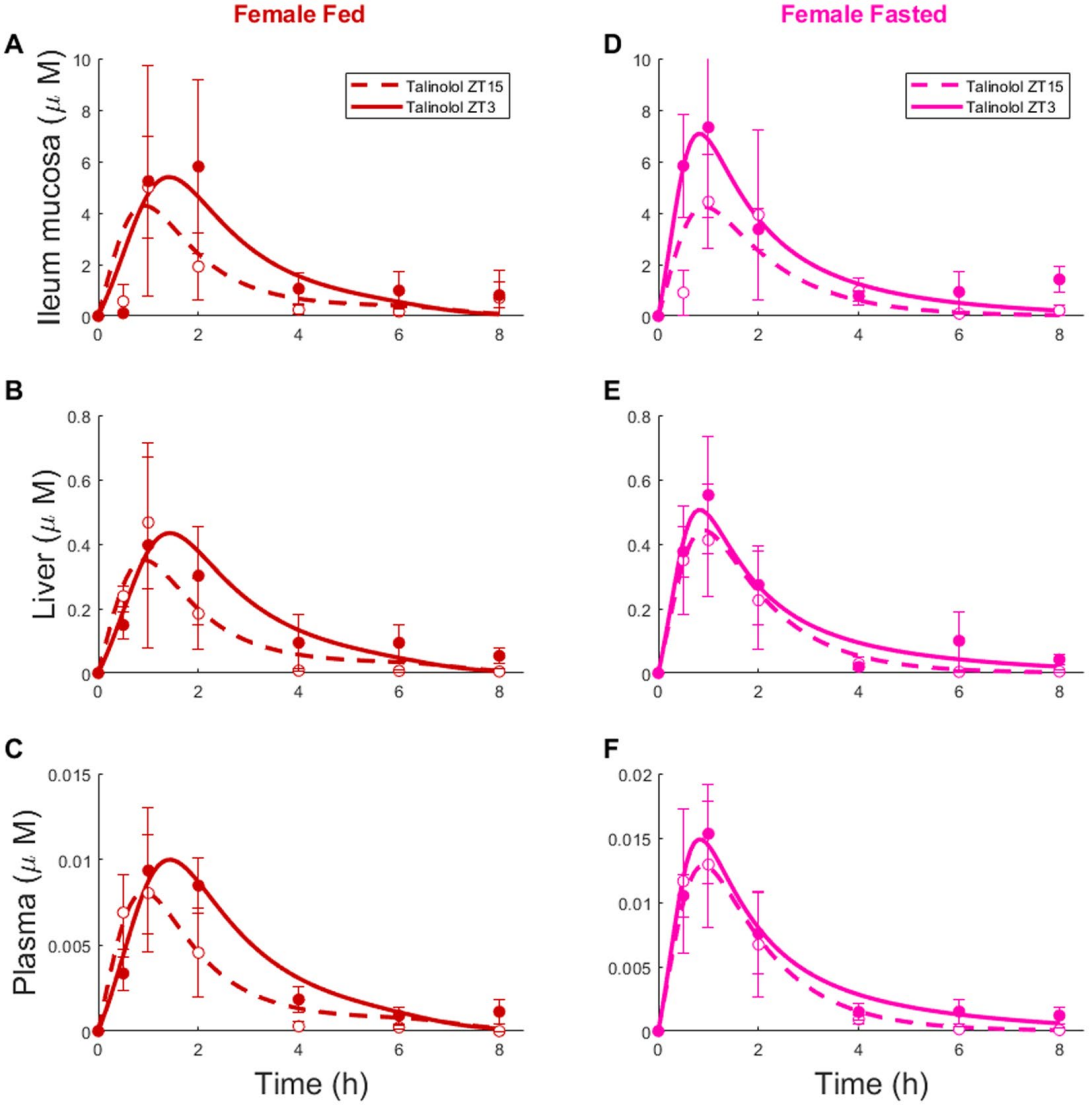
Talinolol ChronoPK in female mice. Concentration-time profiles in *ad libitum* fed (**A**–**C**) or fasted (**D**–**F**) mice after drug oral administration at ZT3 or ZT15. Each data point represents the mean ± SEM of 4 to 5 mice. Solid and dashed curves are the model best-fit to data points.

**Figure 4:**
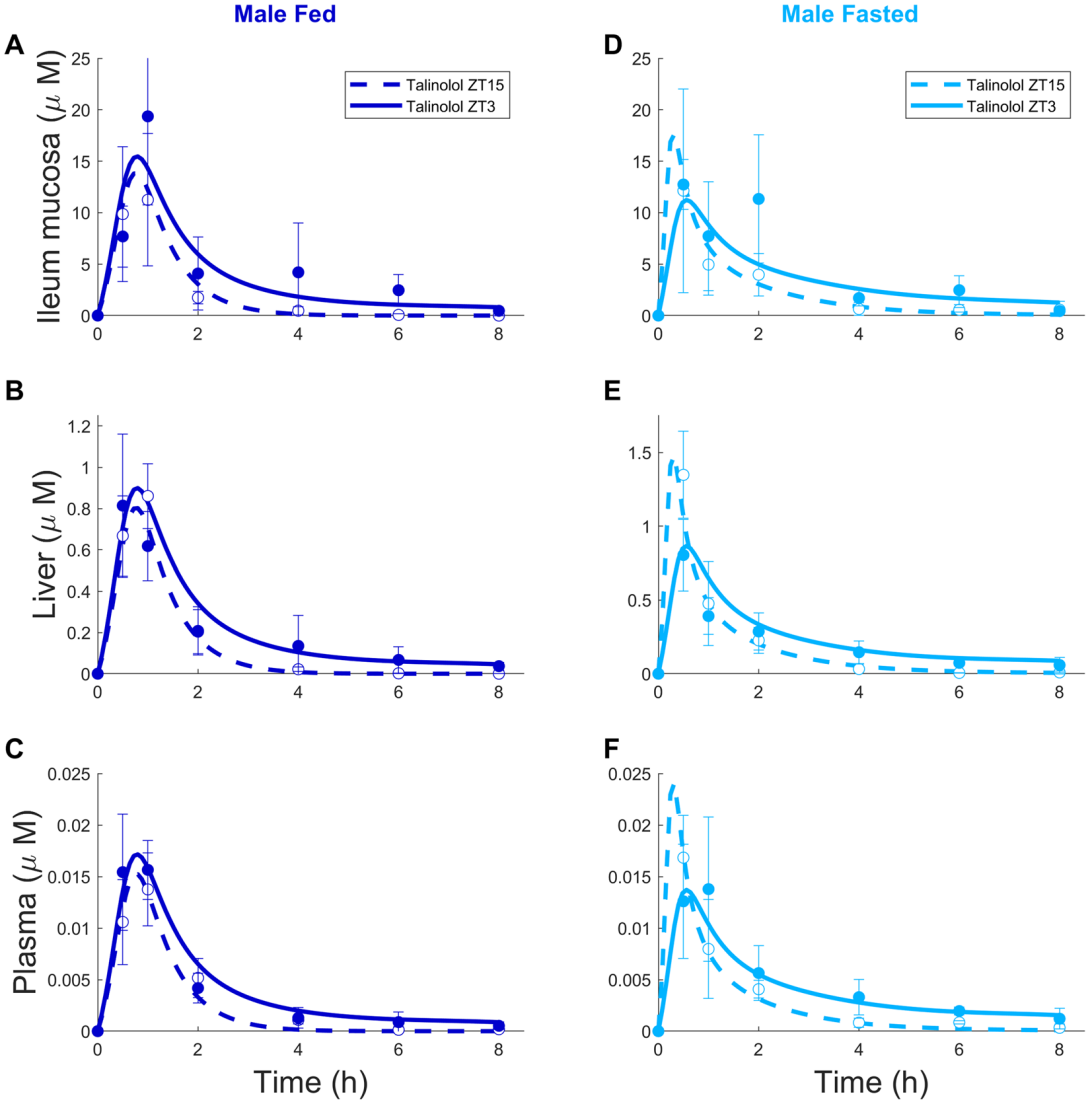
Talinolol ChronoPK in male mice. Concentration-time profiles in *ad libitum* fed (**A**–**C**) or fasted (**D**–**F**) mice after drug oral administration at ZT3 or ZT15. Each data point represents the mean ± SEM of 4 to 5 mice. Solid and dashed curves are the model best-fit to data points.

Talinolol was used to investigate *P-gp* activity at whole organism level in mice of both sexes, either *ad libitum* fed or fasted, following dosing at one of two circadian times (Figs 3 and 4). In all experimental groups and in all organs, talinolol concentrations reached maximum levels 30 min to 1 h after drug administration. A second peak in talinolol concentration was observed 2 h after drug administration only in the ileum of fasted male mice. Talinolol concentrations were highest in mouse ileum, with liver and plasma levels being 10 and 100 times less respectively. Area Under the Curves (AUC) and maximum values (C_max_) of time-concentration profiles were smaller in females than in males, irrespective of organ, feeding condition or dosing ZT (Table S4). AUC values were consistently smaller at ZT15 compared to ZT3 in all conditions, except in the liver of fasted male mice. Four-way ANOVA testing organ, sex, feeding status and ZT of administration revealed statistically significant differences in AUC and C_max_ values (p < 0.0001) for all factors and interactions.

### Physiologically-based model as a tool for experimental design

**Figure 5:**
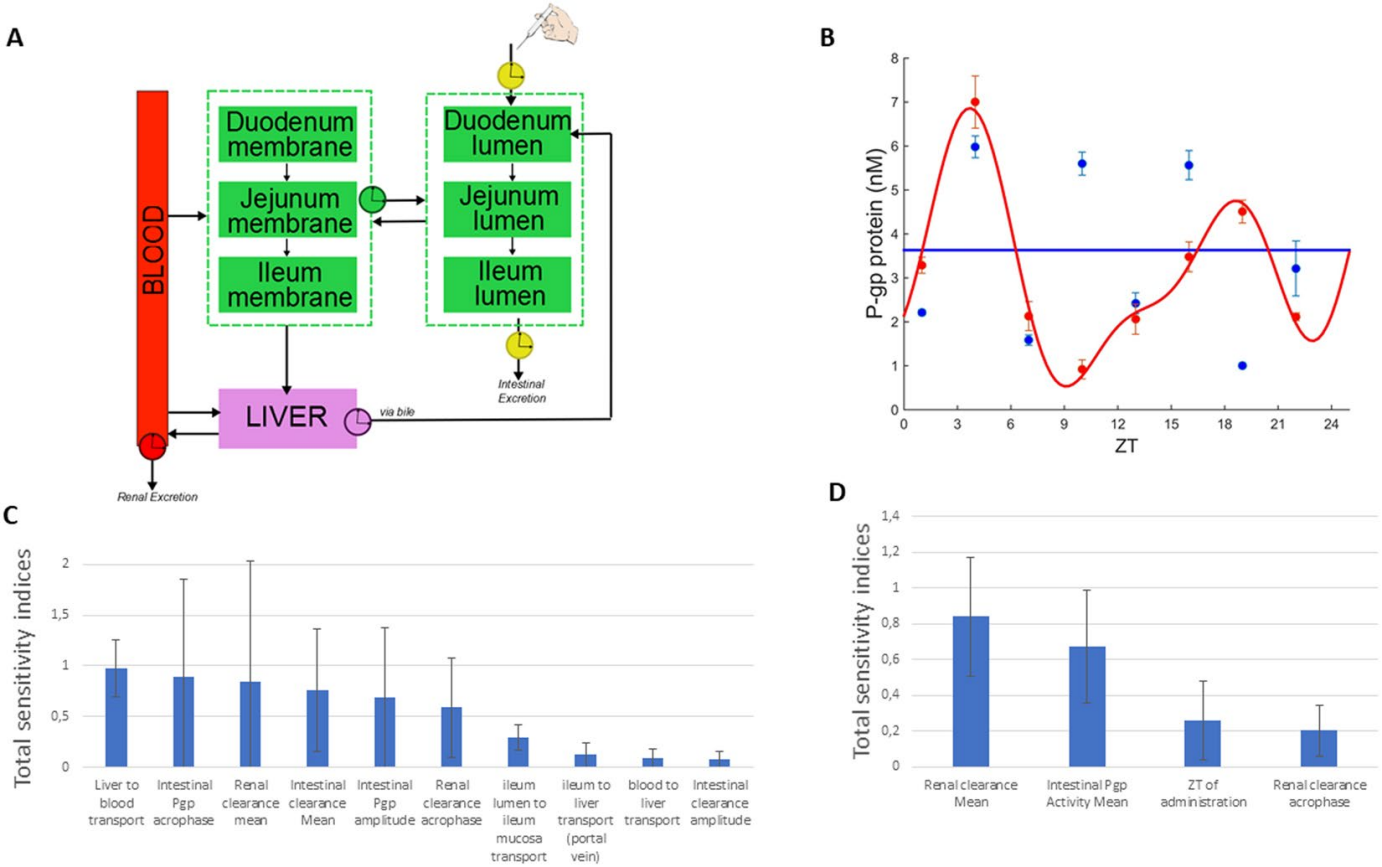
Physiologically-based model of talinolol PK. (**A**) Physiologically-based model of talinolol chronoPK, (**B**) P-gp protein expression in the liver of female (red) and male (blue) mice and best-fit cosinor (**C**–**D**) Global sensitivity of model parameters to talinolol AUC_0–8h_ in the liver (**C**) and the ileum (**D**).

The physiologically-based model of talinolol chronoPK was calibrated to both ileum *P-gp* protein and talinolol PK datasets, so as to analyze *P-gp* activity at whole organism level (Fig. 5A). A set of parameters was estimated for each group, male or female, and feeding condition, *ad libitum* fed or fasting. For fed mice, ileum *P-gp* circadian periods, amplitudes and phases were set to those estimated from protein data (Fig. 1). For fasted mice, circadian periods were set to those found for fed animals of the same sex (24 h for females, 12 h for males) and corresponding circadian amplitudes and phases were estimated from PK datasets. Circadian phases of renal and intestinal clearance were assigned to occur during the nocturnal active phase, i.e. between ZT12 and ZT24^38^. Amplitudes in fasted mice were assumed to be less than those in *ad libitum* fed animals as fasting decreased *P-gp* expression circadian amplitudes compared to *ad libitum* feeding^39,40^. This first parameter estimation procedure failed to predict reliably hepatic *P-gp* circadian activity. Sensitivity analysis on model parameters could not rule out an important contribution of hepatic *P-gp* rhythms to the whole-organism *P-gp* detoxification function. Hence, we experimentally determined the 24 h patterns of *P-gp* protein level in the liver of *ad libitum* fed male and female mice (Fig. 5B).

### Sex-specific circadian changes in *P-gp* levels in liver

The expression of liver *P-gp* protein displayed circadian changes in females with a major peak at ZT4 and a secondary peak at ZT20 (Fig. 5B). A predominant 24 h-rhythm and significant 12 h and 8 h harmonics were identified with cosinor analysis for females (p = 0.024) but not for males (p = 0.23). Two-way ANOVA tests statistically validated the effect of ZT (p < 0.0001) and of ZT*sex (p < 0.0001), but not that of sex alone (p = 0.53) in these datasets. Twenty-four-hour mean values were similar in both sexes (Table S3).

### *P-gp* activity as predicted by physiologically-based analysis of multi-type data

**Figure 6:**
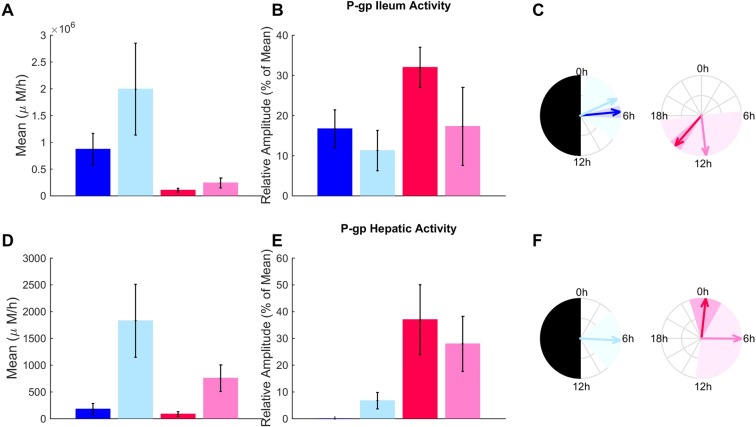
Ileum and hepatic *P-gp* activity according to sex, feeding and circadian timing. Mesors, amplitudes and phases (averages ± SD) of P-gp activity estimates from physiologically-based analysis of P-gp protein and talinolol PK data are shown in fed and fasted male mice (dark and light blue respectively) and in fed and fasted females (dark and light pink respectively). Circadian amplitudes and phases of P-gp activities in fed animals were directly derived from the cosinor analysis of protein data (panels B, C, E and F). P-gp hepatic activity in *ad libitum* fed male mice did not display significant circadian rhythms so that the amplitude was equal to 0 and no phase could be computed.

A second parameter estimation procedure was run, that now integrated the hepatic *P-gp* protein data (Fig. 6 and Supplementary data). This allowed to compute optimal parameter values and standard deviations for each of the 4 mouse categories. Both sex and feeding conditions significantly influenced estimated circadian mesors, amplitudes and phases of both intestinal and hepatic *P-gp* activity (two-way ANOVA significant for all P-gp related parameters).

Regarding sex–related differences, the model-estimated mesors of *P-gp* activity were higher in male compared to female ileum and liver, in both feeding conditions. Talinolol passive diffusion rates from the intestinal lumen to intestinal cells were also faster in males compared to females (Fig. S3B). Model-estimated circadian amplitudes were consistently larger in females as compared to males. Circadian phases also significantly varied by up to 10 h with respect to sex.

The model predicted fasting to increase *P-gp* activity in both liver and ileum in male mice, which was in agreement with prior reports^7,8^, thus providing a partial model validation. All model-derived circadian amplitudes were decreased in fasted mice compared to *ad libitum* fed animals. No conclusion could be drawn regarding the effect of fasting on circadian phases as the estimated parameters presented large standard deviations (Fig. 6C,F).

### Sex-, feeding and circadian time-specific talinolol elimination as predicted by physiologically-based analysis

**Figure 7:**
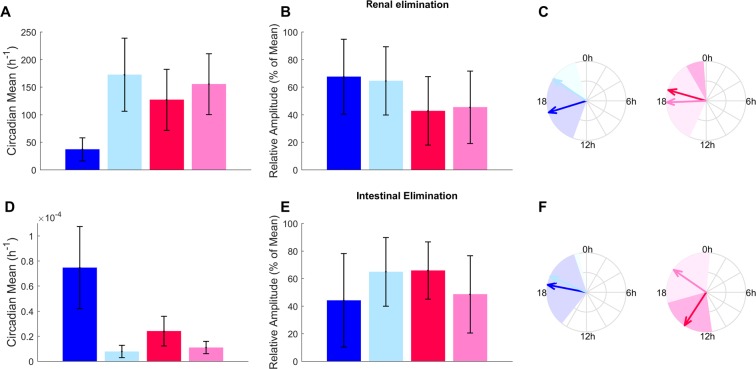
Talinolol renal and intestinal elimination according to sex, feeding and circadian timing. Mesors amplitudes and phases (averages ± SD) of P-gp activity estimates from physiologically-based analysis of P-gp protein and talinolol PK data are shown in fed and fasted male mice (dark and light blue respectively) and in fed and fasted females (dark and light pink respectively).

The mathematical model was utilized to analyze the circadian changes in talinolol elimination in the 4 mouse classes (Fig. 7). The model predicted that renal excretion was by far the main route of elimination with transport rates being 5 orders of magnitude greater compared to intestinal clearance, in good agreement with the scientific literature, thus providing once more a partial model validation^22^. Two-way ANOVA validated sex- and feeding-related differences in model-estimated 24 h-mean of renal elimination (sex* feeding p < 0.001). Highest renal eliminations were predicted in fasting conditions. Model-estimated amplitudes of renal excretion rhythms differed according to sex with slightly smaller values for females compared to males. Regarding intestinal elimination, the model-derived 24 h-means significantly differed according to both sex and feeding conditions (p < 0.001), and were least in fasted animals, possibly resulting from the reduced gastric motility in the absence of food^41^. No difference was validated for circadian amplitudes according to sex or feeding condition, but acrophases varied according to both factors (ANOVA, p < 0.001). Model-estimated renal and intestinal elimination displayed relative circadian amplitudes of approximately 50% with circadian phases occurring between ZT14 and ZT20 consistently with previous results, thus providing a further model validation^38,42–44^.

### Determinants of intestinal and liver talinolol PK

Sensitivity analysis were performed to determine the key model parameters driving talinolol tissue concentrations for all mouse categories (Fig. 5C,D). Talinolol concentration in the ileum was mainly determined by 4 quantities: 24 h-mean and phase of renal elimination, 24 h-mean of ileum *P-gp* activity and circadian time of administration. On the opposite, talinolol liver concentration was determined by multiple parameters including liver-to-blood transport rate, ileum *P-gp* 24 h-mean, amplitude and phase, and renal elimination 24 h-mean and phase. Altogether, these results confirmed the minimal influence of intestinal clearance and highlighted the critical relevance of active intestinal *P-gp* activity and passive renal elimination, and their circadian rhythms for talinolol tissue PK.

### *Ex-vivo* P-gp expression in ileum mucosa

**Figure 8:**
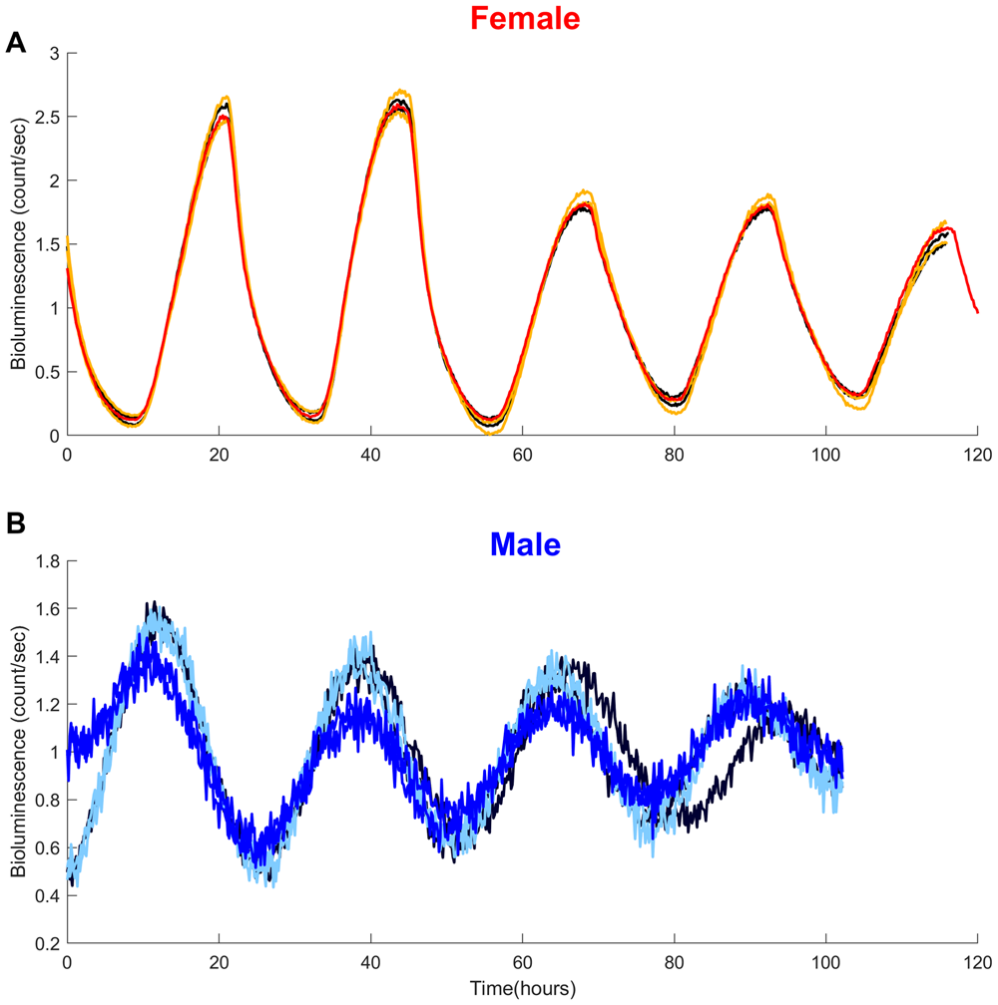
Sex-specific endogenous circadian rhythms in *Abcb1a* reporter expression in *ex-vivo* ileum mucosa samples. Detrended bioluminescence timeseries recorded from 3 different female (**A**) and male (**B**) mice. Duplicates from the same animal are represented in the same color. Circadian rhythms were statistically validated for all individual timeseries.

To further investigate the endogenicity of P-gp circadian rhythms and their sex-specificity, P-gp expression was quantified *ex-vivo* in ileum mucosa samples isolated from male and female *Abcb1a-luc* reporter mice because *Abcb1a* is the closest murine homologue of the human ABCB1 gene, which codes for P-gp^45^ (see Methods, Fig. 8). Significant circadian rhythms were found for both sexes with a period of 26 h 35 ± 38 min (SD) for males and 24 h ± 3 min for females (p < 0.001). Maximum amplitudes relative to mesor were larger in females as compared to males with mean values (±SD) equal to 127% ± 6% and 51% ± 12% respectively. Circadian phases (±SD) were located at 19 h 13 ± 3 min for females and at 11 h 32 ± 57 min for males so that their respective values in radians differed by 37% of 2π. The dampening parameter was smaller in females (0.0054 ± 0.0004 h^−1^) as compared to males (0.0096 ± 0.001 h^−1^). Inter- and intra-individual variabilities were slightly larger in males as compared to females although they were remarkably low in this experiment (Fig. 8).

## Discussion

*P-gp* can critically affect whole-body PK of many medications including numerous anticancer agents, thus driving drug tolerability. Hence, predicting *P-gp* dynamics within individual subjects could allow for dosing schedule personalization in order to minimize side effects. The current investigation revealed that this was indeed a multi-factorial challenge, as *P-gp* expression and activity largely varied according to organ, sex, feeding status and circadian time, in an inter-related manner. Hence, quantitative systems pharmacology appeared mandatory to jointly address the issue of P-gp substrates dose and timing according to sex and feeding. The data-driven mathematical model that was developed successfully recapitulated multi-type experimental datasets and provided educated model predictions. The model was further validated through its good agreement with independent data reported in scientific literature. Importantly, the analysis of each dataset separately would not have allowed us to estimate P-gp activity according to the multiple studied factors.

*P-gp* mRNA and protein levels were rhythmic in the ileum, which is a major organ for drug absorption. An *ex-vivo* study of organotypic slices demonstrated – for the first time -endogenous circadian rhythms of P-gp protein expression in ileum mucosa in the absence of external synchronizer or internal time cue. On the opposite, *P-gp* expression did not show major time-dependent variations in the colon. This was in agreement with a recent *in vitro* study in human colorectal Caco-2 cells that concluded that *P-gp* circadian rhythms played a minor role in the chronotoxicity of irinotecan which was mostly driven by the rhythms in drug bioactivation and metabolite deactivation^16^.

A second peak in talinolol time-concentration profiles was observed 2 hours after drug administration in fasted male mice treated at ZT3. This double-peak phenomenon has been found in talinolol PK studies in patients^46,47^. Enterohepatic recirculation is the classical explanation for such double-peak PK but it may not be relevant for talinolol which displays limited reabsorption^46,48^. A possible explanation rather involves a pre-systemic drug processing through talinolol binding to bile acids in the intestinal lumen as demonstrated for pafenolol in fasted rats^48,49^. Indeed, pafenolol formed micellar complexes with bile acids in the proximal small intestine, which dissociated in the ileum, thus enabling pafenolol ileum absorption and its translation into the second peak in plasma drug concentration. This phenomenon was not present in *ad libitum* fed animals as food may prevent the formation of such complexes^49^.

Large sex-related differences were observed in intestinal and hepatic *P-gp* activity 24 h-means, which were estimated to be higher in males compared to females. Circadian amplitudes of *P-gp* mRNA and protein level rhythms were consistently higher in females than in males in all organs and in all feeding conditions. The cellular basis of the sex-related differences in circadian P-gp protein was further highlighted through continuous bioluminescence reporter monitoring and time series analysis of *ex vivo* ileum mucosa samples from Abcb1a-luc mice. Indeed, the endogenous period of the bioluminescent protein was significantly shorter, the relative circadian amplitude was larger, and the acrophase occurred later in females as compared to males. Prior studies showed significantly larger circadian amplitudes in rest-activity and plasma corticosterone in female, as compared to male mice of the same strain and with similar age as those used in the current study^26^. This was also the case for the circadian mRNA expressions of clock genes Rev-erbα, Per2, and Bmal1 in the liver of female as compared to male mice^27^. Further, recent preclinical and clinical investigations showed higher circadian amplitudes in females compared to males in the chronotoxicity of the anticancer drug irinotecan, which is a P-gp substrate^27,50^. Differences in feeding patterns between males and females could also play a role in the sex-specific variations demonstrated here for P-gp expression and activity since food intake was reported to be larger in females as compared to male C57Bl/6 mice^51^. The fasted mice receiving talinolol at ZT3 were fasted from ZT15 to ZT3 and remained fasted for up to 8 h, for the duration of talinolol PK experiments, i.e. until ZT 11. The fasted mice receiving talinolol at ZT15 were fasted from ZT3 to ZT15, then for up to 8 h until ZT23. Hence, fasting time was the same in both subgroups, but the fasting circadian window was different, which could represent a limitation of our study.

An important motivation for this work was the design of a model of irinotecan chronoPK-PD for the personalization of the drug chronotherapy, including sexual dimorphism in toxic response to treatments^4^. The clinical relevance of sex-related toxicities has been demonstrated for the hematologic or mucosal toxicities of anticancer drugs 5-fluorouracil, oxaliplatin, or irinotecan. In these large studies, women displayed higher toxicity rates by up to 15% as compared to men^9,52–54^. Consistently with these findings, a data mining analysis of global liver safety reports was conducted for 375 drugs associated with hepatotoxicity, from WHO VigiBaseTM^55^. A higher overall liver event reporting for women and a higher reporting of acute liver failure for younger women were identified^56^. Forty-one drugs with female-biased reported frequencies were associated with a higher prevalence of reactive metabolite formation and mitochondrial liability, and higher ABCB11 and ABCC2 transporter inhibition potential. Moreover, our studies further highlighted that the tolerability of irinotecan, like that of oxaliplatin, largely varied according to sex and circadian timing in mice and/or in humans^4,57^.

A mathematical model of irinotecan chronoPK-PD has been developed but model calibration was not possible due to the large number of parameters to be estimated simultaneously^58^. Importantly, the present study has provided estimates for the circadian amplitudes and phases of P-gp hepatic and ileum circadian activity in B6D2F1 male and female mice, either *ad libitum* fed or fasted. Those parameter values can be directly incorporated into the irinotecan PK-PD model. This is a critical step towards the complete calibration of the model, that will then be used for optimizing irinotecan chronotherapy for each mouse category. The model will also assist in the identification of key circadian determinants of irinotecan chronotoxicity. Interestingly, the circadian rhythms in P-gp activity that were identified in this study are likely to be mainly driven by the amount of active P-gp molecules within the cell membrane and thus to be drug-independent. Hence, the estimated circadian amplitudes and phases of P-gp hepatic and intestinal activity can be incorporated into PK models of any *P-gp* substrate studied in B6D2F1 mice. Hence, this study has provided all the mathematical components- equations and parameter estimates- needed to extend classical physiologically-based PK models to incorporate sex-specific circadian rhythms.

Our study lays the grounds for further human application. Such PK-PD model holds the potential to be scaled for humans to predict optimal drug dose and timing in individual patients^4^. From a general standpoint, physiologically-based modelling allows for multiscale chronoPK-PD approaches aiming to design human models based on both preclinical and clinical data. Mouse studies serve as a basis to design the drug-specific structure of the whole-body PK-PD model, which incorporates the drug pharmacology in each considered organ. Next, a model for an average patient can be obtained by keeping the structure of the mouse model and resizing the parameters for humans using physiological literature information combined to clinical datasets in patient populations. The generic model can then be informed with patient-specific features such as the patient’s sex, chronotype or P-gp polymorphisms, to ultimately compute personalized dose and administration schedules. This model translation from preclinical studies to humans holds great promises towards personalized and precision medicine^4^.

## Methods

### Animals and synchronization

Male and female B6D2F1 mice (5 to 7 weeks of age, Janvier, Le Genest St Isle, France) or heterozygous B6;129S1-Abcb1a^tm1.1Kane^ mice^45^ (crossing of heterozygous C57B/L6 male and homozygous B129 mice, referred to as Abcb1a-luc mice thoughout the text, 8–10 weeks of age, a kind gift from Susan Kane, Beckman Research Institute, City of Hope, Duarte, CA 91010-3000, USA) were held in temperature controlled (23 ± 1 °C), ventilated (100 ± 10 l/min) and light tight boxes with 12 h of light (L) alternating with 12 h of darkness (D) (LD12:12) and *ad libitum* access to food and water. Animals were housed in the controlled room for three weeks before starting the experiments. Dim red light (7 lux) was used for manipulations during the dark phase. Time of day is referred as Zeitgeber Time (ZT) with ZT0 corresponding to light onset. Whenever mice were fasted, food access was withdrawn 12 h before the first experimental intervention. All experimental protocols were conducted following the guidelines for animal experimental procedures issued by the French Ethical Committee, decree 87–848 and the European Communities Council Directive of 24/11/1986 (86/609/EEC). The experimental protocols were approved by the experimental animal sub-committee of the Andre Lwoff Institute (Villejuif, France) or by the Home Office (UK, Project Licence 7008536).

### Compounds and reagents

Talinolol and escitalopram were generously given by Arzneimittelwerk Dresden (AWD Pharma, Radebeul, Germany) and Neutec Pharmaceutical Co. (Istanbul, Turkey) respectively. Buffer salts, solvents and reagents were purchased from E. Merck (Darmstadt, Germany) unless stated otherwise.

### mRNA expression studies

*Abcb1a/b* mRNA expression were determined with RT-qPCR^25^. Samples were immediately frozen in liquid nitrogen, then stored at −80 °C. Total RNA was extracted from frozen specimens by liquid-liquid extraction. Each sample was first homogenized with 2 mL lysis buffer GIT (guanosine isothiocyanate), then sodium citrate and N-lauryl sarcosine 10%, 2-mercaptoethanol were added. The lysate was recovered in a tube for RNA^59^. Next, phenol and chloroform isoamyl (24:1, v/v) were added. After agitation, the samples were incubated for 15 min on ice and then centrifuged (20 min, 9660 g at 4 °C). The aqueous phase was then recovered. One volume of isopropanol was added, and the mixture was obtained through iterative reversals of the solution. Precipitated mRNAs were kept overnight at −80 °C. On the next day, samples were centrifuged (30 min, 9660 × g, at 4 °C) and supernatants were removed. The pellets were rinsed with ethanol 70% (500 µl), then centrifuged (20 min, 9660 × g at 4 °C). The supernatants were discarded, and pellets dried for 10 min on ice, then re-suspended in RNA-free water. RNA-free samples were analyzed by formaldehyde gel electrophoresis, and integrity was confirmed by visualization of 18S and 28S rRNA bands. The RNAs obtained were quantified by spectrometry (Biophotometer Plus, Eppendorf, France) and stored −80 °C.

Reverse transcription involved a denaturating step of RNA, followed by the retro-transcription step (RT Invitrogen, Cergy-Pontoise, France). The thermocycler was used for all stages of heating and cooling. For each sample, a solution containing 5 µg of total RNA and 100 ng of random primers (hexamers solution at 100 ng/µl) was prepared. It was then heated in the thermocycler at 65 °C for 5 min (denaturation step), then it was cooled at 4 °C for 5 min. Along with the denaturing step, the solution required for the retro-transcription was prepared. It contained desoxynucleoside triphosphates (dATP, dCTP, dTTP and dGTP) (10 mM), a buffer containing the enzyme cofactors essential for enzyme function (250 mM Tris-HCl, 375 mM KCl, 15 mM MgCl_2_), dithiothreitol (0.1 mM solution), and reverse transcriptase (SuperScript II Reverse Transcriptase, Invitrogen, Cergy-Pontoise, France). For the reverse transcription step, samples were incubated for 50 min at 42 °C, and then for 15 min at 75 °C for enzyme denaturation. Next, samples were diluted to the 10^th^ and the PCR was performed using a Light Cycler 3 (Roche Applied Science, Meylan France) with SYBR green I dye detection. A reaction mixture (Faststart DNA SYBR Green I; Roche Diagnostics, Meylan France) was supplemented with cDNA and primers (0.5 µM). A standard curve was generated to compute relative mRNA abundance. mRNA expression levels were normalized to the amount of the constitutively and non-rhythmically expressed 36B4 (acidic ribosomal phosphoprotein)^25^.

### *P-gp* protein expression in ileum and colon mucosa

Ileum and colon mucosa samples were analysed as described in^25^. Briefly, samples were fixed in buffered formalin solution (4%), kept at room temperature for 24 h, dehydrated in ethanol, cleared in Bioclear (Bioptica, Milan, Italy) and embedded in paraffin. Five- µm sections were transferred to positively charged slides which were incubated overnight at 37 °C and immersed in xylene. Tissue sections were rehydrated (graded alcohol series 100%, 96% and 80% for 5 min each and rinse in water) and stained with haematoxylineosin. Immunohistochemistry (IHC) analysis was performed as described in^25^ using a mouse anti-mouse *P-gp* antibody (Covance, Princeton, NJ, USA; diluted 1/20) and a rabbit anti-mouse *β-catenin* antibody (Neomarkers, Fremont, CA, USA; diluted 1/50). Three images were collected for each tissue section corresponding to β-catenin positive areas as this latter protein is co-localized with P-gp at the intestinal mucosa. Image analysis was performed as described in^25^.

### Talinolol chronoPK study

Talinolol was suspended in methylcellulose (0.5%) and administered via oral gavage (100 mg/kg). Controls received only vehicle, i.e. 0.5% methylcellulose solution. A single dose of talinolol was administered to 60 male and 60 female mice at ZT3 or ZT15. Those times of administration were chosen according to previous results in male mice^18,20^ or rats^22^ and are associated to low (ZT3) and high (ZT15) intestinal P-gp activity. The study involved 4 or 5 mice of each sex sacrificed 30 min, 1, 2, 4, 6 and 8 h after talinolol administration. Mice were sacrificed and blood samples (~1 mL each) were drawn on heparin from the retro-orbital sinus. Blood samples were immediately centrifuged at 12,000 rpm for 10 min at 4 °C and plasma was separated. Liver and ileum mucosa were sampled immediately following sacrifice. Ileum samples were cut longitudinally, washed with isotonic saline solution and dried gently. The mucosa layer was scraped with a scalpel. All samples were kept at −25 °C until analyses.

### Determination of talinolol in plasma

The analysis of plasma talinolol was carried out with High Performance Liquid Chromatography (HPLC), using Waters 2695 pump, autosampler, column heater-sample cooler (Waters, Milford MA, USA) and Waters 2487 UV/VIS detector (Waters, Milford MA, USA)^22^. Talinolol was extracted from plasma by hydrophilic-lipophilic balance type solid phase extraction cartridges (Oasis^®^ SPE cartridges 1 cc/30 mg; Waters, Milford MA, USA) and reversed phase Phenomenex C_18_ column (250 × 4,6 mm; 5 µm, Waters, Milford MA, USA) connected with a universal Phenomenex C_18_ precolumn (2,1 × 3,9 mm; 5 µm, Varian-USA). The extraction was carried out using a dedicated sample preparation product (VacElut, Varian, Palo Alto CA, USA) and a vacuum pump (KNF, Freiburg, Germany). Firstly, cartridges were conditioned with 1 mL HPLC grade methanol and 1 mL HPLC grade water (Millipore, Milford MA, USA), then 0.25 mL plasma were passed through the cartridges. Cartridges were rinsed with 1 mL HPLC grade water and water:methanol (95:5, v/v) for unretained matrix, then talinolol was collected in clean tubes by eluting with 0.5 mL methanol (20 µl). A mixture of 0.05 M phosphate buffer/acetonitrile (73:27, v/v; pH: 4) was used as a mobile phase for HPLC. Flow rate was 1 mL/min, the column temperature was adjusted to 40 °C and wavelength eluent monitoring was 242 nm. Retention time of talinolol was 9.5 min and the limit of determination was 50 ng/mL. Intra-day reproducibility was 6.1% for 1 µg/mL and 4.5% for 0.25 µg/mL. Inter-day reproducibility was 6.5% and 2.9% for 1 and 0.25 µg/mL, respectively.

### Determination of talinolol in liver and ileum mucosa

Liver and ileum mucosa samples were homogenized using 0.9% NaCl in water (1:5–1:10 ratio w:v) with UltraTurrax (Germany) 2 min for liver, 30 sec for ileum mucosa, and vortexed for 1 min for both tissues. Talinolol was determined in homogenized tissue aliquots (200 µl) using the same HPLC-UV/VIS detector^60^. Talinolol was extracted by liquid-liquid extraction using 2 mL ter-methyl butyl ether, following addition of internal standard (IS), 10 µl (4,5 µg/mL) escitalopram (Neutec Pharmaceutical Co., Istanbul, Turkey) and 200 μl of 0.5 M NaOH. After addition of all reagents, the mixture was vortexed and, centrifuged at 2500 g for 2 min. The organic layer was collected in a clean tube and evaporated in a block heater at 55 °C (Stuart, UK). The samples were reconstituted in 200 µL of mobile phase and analyzed by HPLC on a Phenomenex column (250 × 4 mm; 5 µm, Varian, USA) connected with a universal Phenomenex C_18_ precolumn (4 × 4 mm; 5 µm, Varian). Mobile phase contained 0.05 M phosphate buffer/acetonitrile (66:34, v/v; pH: 3.1) at a flow rate of 1 mL/min with column temperature set at 35 °C, the injection volume was 45 μL and wavelength for eluate monitoring was 245 nm. Triethylamine was used as peak modifier (0.6 mL per liter). Retention time of talinolol was 4.4 min and retention time of IS escitalopram was 6.2 min. Variability of the assay ranged from 2 to 10%, and the limit of determination was 50 ng/mL.

### Liver *P-gp* protein expression

Forty-eight male and 48 female B6D2F_1_ mice were randomized into 8 groups of 6 mice per sex each and entrained to LD12:12 cycles for two weeks. Mouse liver was sampled from separate groups at ZT1, 4, 7, 10, 13, 16, 19, and 22. After flushing the liver with PBS, it was extracted, weighed and immediately frozen and stored till analysis at −80 °C. Liver tissue was homogenized in 1 ml PBS/g of liver on ice by fast rotating homogenizer (T10 basic Ultra-Turrax Homogenizer, IKA, Germany), then subjected to three freeze-thaw cycles. Protein supernatant was collected after centrifugation (15 min, 2500 g, 4 °C). Total protein was quantified using Bradford protein assay (AppliChem GmbH, Darmstadt, Germany) prior to *P-gp* determination. *P-gp* protein content was quantified using ELISA according to the manufacturer’s instructions (Mouse Permeability Glycoprotein Elisa kit, Blue Gene Biotech, Shanghai, China).

### P-gp protein expression in *ex-vivo* ileum mucosa

Six Abcb1a-luc mice (3 males and 3 females) aged 12 weeks were entrained to LD12:12 cycles for 2 weeks prior to experiments^45^. On the day of the experiment, the mice were euthanized by cervical dislocation at ZT3 and ileum mucosa was sampled and stored in ice cold HBSS (Sigma, Dorset, UK). The isolated ileum mucosa was cleaned with HBSS buffer and cut in a longitudinal section to expose the inner lumen. The ileum mucosa from each mouse was divided into two samples to serve as intrasubject replicates. The slices were put on 30 mm polytetrafluoroethylene inserts with a pore size of 0.4 µm. in 35 mm tissue culture dishes (Thermo, Cambridge, UK) and 1.2 mL culture medium (phenol-free DMEM, 10% FBS, 1% Pen/Step, 10 mM HEPES) with 20 µM luciferin. The dishes were sealed with silicon grease and put into a Lumicycle (Actimetrics, Illinois, USA) at constant temperature (37 °C) to measure the bioluminescence as photon counts per min every 10 min for up to 6 days.

### Statistical analysis

Variance arising from organ, sex, feeding condition and circadian timing was tested though multi-factor ANOVA. The statistical significance of a cosine waveform was determined with cosinor analysis testing for the main period of 24 h and the two first harmonics at 12 h and 8 h^61^. Time series involved one datapoint every 3 h for 24 h which theoretically allows for the determination of oscillations with periods above 6 h according to Nyquist theorem^62^. The cosinor method computes the mesor (rhythm-adjusted mean), and, for each harmonic, the double amplitude (difference between minimum and maximum of fitted cosine function), and the phase (time of maximum in best-fitting cosine function). The Area Under the Curve (AUC) over the first 8 h of drug exposure and the maximum concentration Cmax of talinolol PK curves were computed from experimental data as follows. For each mouse dataset, talinolol time-concentration profiles were generated by randomly combining individual mouse PK measurements for each time point. This was done for all possible combinations and yields approximately 5000 curves from which Cmax and AUC values were computed. Means and standard deviations were then inferred for each condition. Regarding the analysis of *ex-vivo* ileum mucosa bioluminescence, a linear trend was removed from time series, which were then normalized to obtain a mean value equal to 1. Then, a damped cosine was fitted to detrended normalized data, using a least-square method, to compute the period, phase, maximum amplitude and dampening parameter^63^. Significance for all tests were set to p < 0.05. SPSS v.16 (IBM, Paris, France) or Matlab (Mathworks, USA) were utilized for statistical tests.

### Model parameter estimation and sensitivity analysis

Parameter estimation consisted in a weighted least-square approach using the Covariance Matrix Evolutionary Strategy (CMAES) algorithm for the minimization task^16,63^. Averaged data were first fitted by iteratively running the estimation procedure and updating initial conditions with the current best-fit parameters until reaching convergence. Next, data variability was integrated by using Monte Carlo simulations. Briefly, virtual datasets of talinolol PK in the ileum, liver and plasma were composed by randomly picking an individual mouse for each time point. Parameters were then estimated for each virtual dataset and averages and standard deviations were computed. Two hundred datasets were necessary to insure convergence for all parameters. For sensitivity analysis, all parameters’ lower and upper bounds were set to 10-fold lesser and greater than their estimated values on averaged data. Forty-eight thousand parameter sets were generated from cross-sampling by Saltelli’s extension of Sobol’s method using the MOEA (Multi-Objective Evolutionary Algorithms) framework (version 2.0). Parameters’ total-order sensitivity indices and their standard deviations were computed using the Sobol analysis of the MOEA framework.

## Supplementary information

Supplementary Information

## References

[1] Efferth T, Volm M (2017). Multiple resistance to carcinogens and xenobiotics: P-glycoproteins as universal detoxifiers. Archives of toxicology.

[2] Wallington M (2016). 30-day mortality after systemic anticancer treatment for breast and lung cancer in England: a population-based, observational study. The Lancet Oncology.

[3] Damia G, Garattini S (2014). The pharmacological point of view of resistance to therapy in tumors. Cancer Treatment Reviews.

[4] Ballesta A, Innominato PF, Dallmann R, Rand DA, Levi FA (2017). Systems Chronotherapeutics. Pharmacological reviews.

[5] Prasad B (2014). Interindividual Variability in Hepatic Organic Anion-Transporting Polypeptides and P-Glycoprotein (ABCB1) Protein Expression: Quantification by Liquid Chromatography Tandem Mass Spectroscopy and Influence of Genotype, Age, and Sex. Drug Metabolism and Disposition.

[6] Bebawy M, Chetty M (2009). Gender differences in p-glycoprotein expression and function: effects on drug disposition and outcome. Current drug metabolism.

[7] Kulkarni SR (2014). Fasting induces nuclear factor E2-related factor 2 and ATP-binding Cassette transporters via protein kinase A and Sirtuin-1 in mouse and human. Antioxidants & redox signaling.

[8] Kok T (2003). Induction of hepatic ABC transporter expression is part of the PPARalpha-mediated fasting response in the mouse. Gastroenterology.

[9] Giacchetti S (2012). Sex moderates circadian chemotherapy effects on survival of patients with metastatic colorectal cancer: a meta-analysis. Ann Oncol.

[10] Ballesta A, Zhou Q, Zhang X, Lv H, Gallo JM (2014). Multiscale design of cell-type-specific pharmacokinetic/pharmacodynamic models for personalized medicine: application to temozolomide in brain tumors. CPT: pharmacometrics & systems pharmacology.

[11] Matthaei J (2016). Low heritability in pharmacokinetics of talinolol: a pharmacogenetic twin study on the heritability of the pharmacokinetics of talinolol, a putative probe drug of MDR1 and other membrane transporters. Genome Medicine.

[12] Davis M (2005). Gender Differences in p-Glycoprotein: Drug Toxicity and Response. Journal of Clinical Oncology.

[13] Cook MB, McGlynn KA, Devesa SS, Freedman ND, Anderson WF (2011). Sex Disparities in Cancer Mortality and Survival. Cancer epidemiology, biomarkers & prevention: a publication of the American Association for Cancer Research, cosponsored by the American Society of Preventive Oncology.

[14] Soldin OP, Mattison DR (2009). Sex Differences in Pharmacokinetics and Pharmacodynamics. Clinical pharmacokinetics.

[15] Rigalli JP, Tocchetti GN, Weiss J (2017). Modulation of ABC Transporters by Nuclear Receptors. Physiological, Pathological and Pharmacological Aspects. Current medicinal chemistry.

[16] Dulong S, Ballesta A, Okyar A, Levi F (2015). Identification of Circadian Determinants of Cancer Chronotherapy through *In Vitro* Chronopharmacology and Mathematical Modeling. Mol Cancer Ther.

[17] Zhang YK, Yeager RL, Klaassen CD (2009). Circadian expression profiles of drug-processing genes and transcription factors in mouse liver. Drug Metab Dispos.

[18] Ando H (2005). Daily rhythms of P-glycoprotein expression in mice. Chronobiology international.

[19] Stearns AT, Balakrishnan A, Rhoads DB, Ashley SW, Tavakkolizadeh A (2008). Diurnal rhythmicity in the transcription of jejunal drug transporters. Journal of pharmacological sciences.

[20] Murakami Y, Higashi Y, Matsunaga N, Koyanagi S, Ohdo S (2008). Circadian clock-controlled intestinal expression of the multidrug-resistance gene mdr1a in mice. Gastroenterology.

[21] Iwasaki M (2015). Circadian modulation in the intestinal absorption of P-glycoprotein substrates in monkeys. Mol Pharmacol.

[22] Okyar A (2012). Circadian variations in exsorptive transport: *in situ* intestinal perfusion data and *in vivo* relevance. Chronobiology international.

[23] Levi F, Okyar A, Dulong S, Innominato PF, Clairambault J (2010). Circadian timing in cancer treatments. Annu Rev Pharmacol Toxicol.

[24] Granda TG (2001). Experimental chronotherapy of mouse mammary adenocarcinoma MA13/C with docetaxel and doxorubicin as single agents and in combination. Cancer research.

[25] Okyar A (2011). Strain- and sex-dependent circadian changes in abcc2 transporter expression: implications for irinotecan chronotolerance in mouse ileum. PloS one.

[26] Ahowesso C (2011). Sex and dosing-time dependencies in irinotecan-induced circadian disruption. Chronobiology international.

[27] Li XM (2013). A circadian clock transcription model for the personalization of cancer chronotherapy. Cancer research.

[28] Levi F (1985). Circadian rhythm in tolerance of mice for the new anthracycline analog 4′-O-tetrahydropyranyl-adriamycin (THP). European journal of cancer & clinical oncology.

[29] Levi F, Blazsek I, Ferle-Vidovic A (1988). Circadian and seasonal rhythms in murine bone marrow colony-forming cells affect tolerance for the anticancer agent 4′-O-tetrahydropyranyladriamycin (THP). Experimental hematology.

[30] Lévi, F., Blum, J., Reinberg, A. & Mathé, G. In *Progress in cancer chemo-immunotherapy*: *proceedings of French-Japanese Conference on Antibiotics in Tumor Pharmacology held in Paris-South University*, *September* 5–6, 1983 (eds Mathé, G. & Umezawa, H.) 25–40 (Japan Antibiotics Research Association, 1984).

[31] Lee C, Raffaghello L, Longo VD (2012). Starvation, detoxification, and multidrug resistance in cancer therapy. Drug resistance updates: reviews and commentaries in antimicrobial and anticancer chemotherapy.

[32] Scheving LE, Scheving LA, Tsai TH, Pauly JE (1984). Effect of fasting on circadian rhythmicity in deoxyribonucleic acid synthesis of several murine tissues. The Journal of nutrition.

[33] Scheving LE, Tsai TH, Scheving LA (1983). Chronobiology of the intestinal tract of the mouse. The American journal of anatomy.

[34] Scheving LA (2000). Biological clocks and the digestive system. Gastroenterology.

[35] Bishehsari F, Levi F, Turek FW, Keshavarzian A (2016). Circadian Rhythms in Gastrointestinal Health and Diseases. Gastroenterology.

[36] Pacha J, Sumova A (2013). Circadian regulation of epithelial functions in the intestine. Acta physiologica (Oxford, England).

[37] Hayashi Y (2010). Influence of a time-restricted feeding schedule on the daily rhythm of abcb1a gene expression and its function in rat intestine. J Pharmacol Exp Ther.

[38] Pons M, Tranchot J, L’Azou B, Cambar J (1994). Circadian rhythms of renal hemodynamics in unanesthetized, unrestrained rats. Chronobiology international.

[39] Longo VD, Panda S (2016). Fasting, circadian rhythms, and time restricted feeding in healthy lifespan. Cell metabolism.

[40] Vollmers C (2009). Time of feeding and the intrinsic circadian clock drive rhythms in hepatic gene expression. Proceedings of the National Academy of Sciences of the United States of America.

[41] Worsøe J (2011). Gastric transit and small intestinal transit time and motility assessed by a magnet tracking system. BMC Gastroenterology.

[42] Hoogerwerf WA (2010). Rhythmic changes in colonic motility are regulated by period genes. Am J Physiol Gastrointest Liver Physiol.

[43] Gschossmann JM (2001). Diurnal variation of abdominal motor responses to colorectal distension and plasma cortisol levels in rats. *Neurogastroenterology and motility: the official journal of the European Gastrointestinal Motility*. Society.

[44] Noh JY (2011). Circadian rhythms in urinary functions: possible roles of circadian clocks?. International neurourology journal.

[45] Gu L (2009). A new model for studying tissue-specific mdr1a gene expression *in vivo* by live imaging. Proceedings of the National Academy of Sciences of the United States of America.

[46] Weitschies W (2005). The talinolol double-peak phenomenon is likely caused by presystemic processing after uptake from gut lumen. Pharmaceutical research.

[47] Terhaag B, Gramatte T, Richter K, Voss J, Feller K (1989). The biliary elimination of the selective beta-receptor blocking drug talinolol in man. International journal of clinical pharmacology, therapy, and toxicology.

[48] Mirfazaelian A, Mahmoudian M (2006). A simple pharmacokinetics subroutine for modeling double peak phenomenon. Biopharm Drug Dispos.

[49] Lennernas H, Regardh CG (1993). Evidence for an interaction between the beta-blocker pafenolol and bile salts in the intestinal lumen of the rat leading to dose-dependent oral absorption and double peaks in the plasma concentration-time profile. Pharmaceutical research.

[50] Levi, F. *et al*. Sex-related differences in circadian-dependent tolerability of Irinotecan added to chronomodulated 5-Fluorouracil, Leucovorin and Oxaliplatin: final results from international randomised time-finding study EORTC 05011 in patients with metastatic colorectal cancer (MCC). In *ESMO 2017 Congress*. (Madrid, Spain, 2017).

[51] Achamrah N (2017). Sex differences in response to activity-based anorexia model in C57Bl/6 mice. Physiology & behavior.

[52] Chansky K, Benedetti J, Macdonald JS (2005). Differences in toxicity between men and women treated with 5-fluorouracil therapy for colorectal carcinoma. Cancer.

[53] Giacchetti S (2006). Phase III trial comparing 4-day chronomodulated therapy versus 2-day conventional delivery of fluorouracil, leucovorin, and oxaliplatin as first-line chemotherapy of metastatic colorectal cancer: the European Organisation for Research and Treatment of Cancer Chronotherapy Group. J Clin Oncol.

[54] Cristina V (2018). Association of Patient Sex With Chemotherapy-Related Toxic Effects: A Retrospective Analysis of the PETACC-3 Trial Conducted by the EORTC Gastrointestinal Group. JAMA oncology.

[55] George N, Chen M, Yuen N, Hunt CM, Suzuki A (2018). Interplay of gender, age and drug properties on reporting frequency of drug-induced liver injury. Regulatory toxicology and pharmacology: RTP.

[56] Adam R (2015). Compared efficacy of preservation solutions in liver transplantation: a long-term graft outcome study from the European Liver Transplant Registry. American journal of transplantation: official journal of the American Society of Transplantation and the American Society of Transplant Surgeons.

[57] Levi F (2007). Implications of circadian clocks for the rhythmic delivery of cancer therapeutics. Adv Drug Deliv Rev.

[58] Ballesta, A., Clairambault, J., Dulong, S. & Lévi, F. In *New Challenges for Cancer Systems Biomedicine*. (Springer, 2012).

[59] Chomczynski P, Sacchi N (2006). The single-step method of RNA isolation by acid guanidinium thiocyanate-phenol-chloroform extraction: twenty-something years on. Nature protocols.

[60] Pathak SM, Musmade PB, Bhat KM, Udupa N (2010). Validated HPLC method for quantitative determination of talinolol in rat plasma and application to a preclinical pharmacokinetic study. Bioanalysis.

[61] Cornelissen G (2014). Cosinor-based rhythmometry. Theoretical biology & medical modelling.

[62] Miller, F. P., Vandome, A. F. & McBrewster, J. *Nyquist-Shannon Sampling Theorem: Aliasing, Sine Wave, Signal Processing, Nyquist Rate, Nyquist Frequency, Sampling Rate, Shannon-Hartley Theorem, Whittaker-Shannon Interpolation Formula, Reconstruction from Zero Crossings*. (Alphascript Publishing, 2010).

[63] Ballesta A (2011). A combined experimental and mathematical approach for molecular-based optimization of irinotecan circadian delivery. PLoS Comput Biol.

