# Supplementary material for: Sex-, feeding-, and circadian time-dependency of P-glycoprotein expression and activity - implications for mechanistic pharmacokinetics modeling
Source: Sci Rep. 2019 Jul 19;9:10505. doi: 10.1038/s41598-019-46977-0 (PMC6642159; doi:10.1038/s41598-019-46977-0)
Supplement: Supplementary file 1 — Supplementary Information [file 41598_2019_46977_MOESM1_ESM.pdf]

Supplementary Information

Title

Sex-, feeding-, and circadian time-dependency of P-glycoprotein expression and activity - implications for mechanistic pharmacokinetics modeling.

Running title Sex-specific circadian activity of *P-gp*: a mechanistic study

Authors and Affiliations

Alper Okyar[1*], Swati Kumar[2]*, Elisabeth Filipski[3], Enza Piccolo[4], Narin Ozturk[1], Helena Xandri-Monje[2], Zeliha Pala[1], Kristin Abraham[2], Ana Rita Gato de Jesus Gomes[2], Mehmet N. Orman[5], Xiao-Mei Li[3], Robert Dallmann[2], Francis Lévi[2,3‡], Annabelle Ballesta[2,3‡].

[1]Department of Pharmacology, Istanbul University Faculty of Pharmacy, Beyazit, Istanbul, TR-34116, Turkey.

[2]Division of Biomedical Sciences, Warwick Medical School, University of Warwick, UK.

[3]INSERM and Paris Sud university, UMRS 935, Team "Cancer Chronotherapy and Postoperative Liver", Campus CNRS, Villejuif, F-94807, France.

[4]Università degli Studi G. d'Annunzio Chieti e Pescara, Institute for Advanced Biomedical Technologies, Chieti, Italy

[5]Department of Biostatistics and Medical Informatics, Faculty of Medicine, Ege University, Bornova, Turkey.

* These authors equally contributed to the work

‡These authors equally supervised this work.

Corresponding author: Annabelle Ballesta, INSERM and Paris Sud university, UMRS 935, Campus CNRS, Villejuif, F-94807, France. a.c.ballesta@warwick.ac.uk.

1. Immunohistochemistry study of P-glycoprotein protein expression in mouse ileum and colon

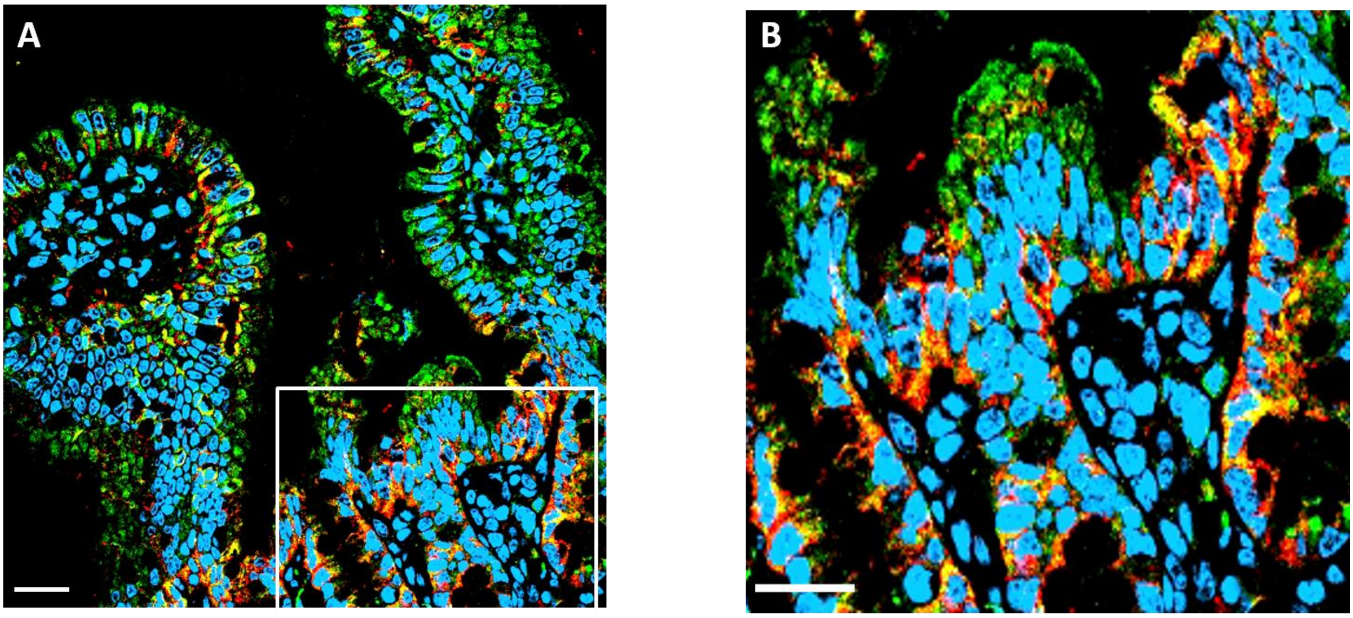

Figure S1 : ***Co-localization of P-glycoprotein and beta-catenin in the membrane of ileum mucosa cells in female mice at ZT15.*** *Double immunofluorescence staining of P-glycoproteein (green) and beta-catenin (red) in mouse ileum using confocal laser scanning microscopy. Co-localization was shown in yellow. Far-red fluorescent DNA dye DRAQ5 (blue) was used to visualize nuclei. The scale bars represent 20 µm.* ***A****. Original magnification 40X (scale bar,).* ***B****. Higher magnification of selected area.*

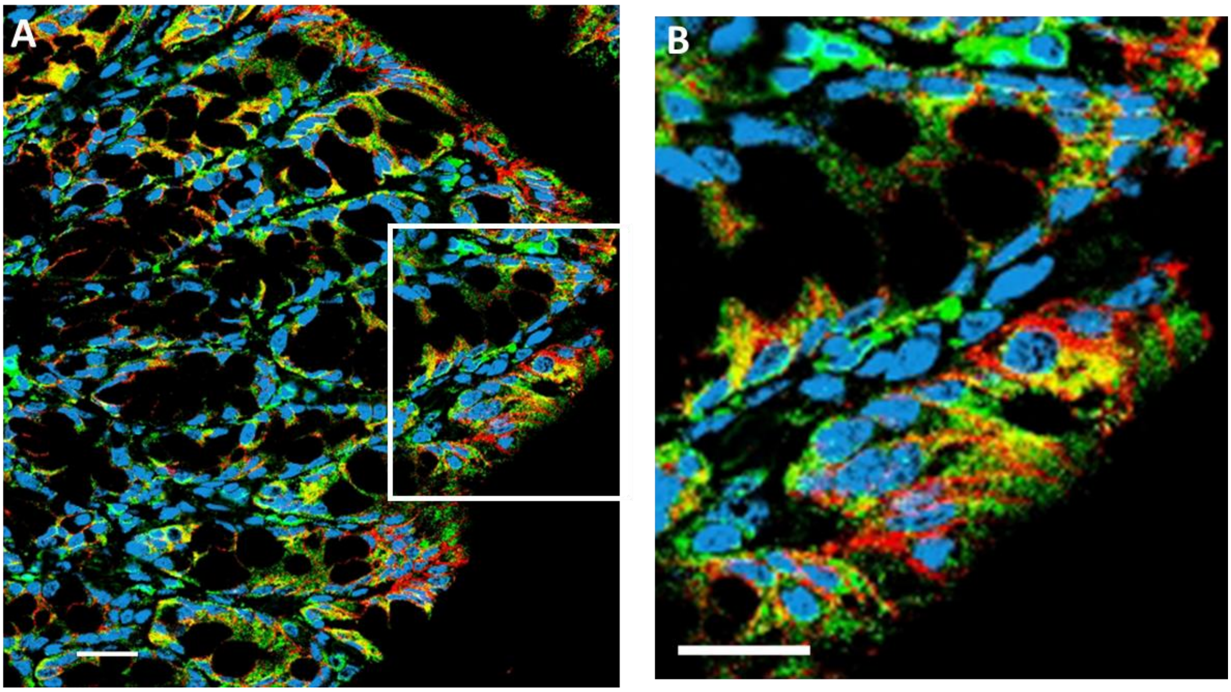

Figure S2: ***Co-localization of P-glycoprotein and beta-catenin in the membrane of colon mucosa cells in female mice at ZT15.*** *Double immunofluorescence staining of P-glycoproteein (green) and beta-catenin (red) in mouse ileum using confocal laser scanning microscopy. Co-localization was shown in yellow. Far-red fluorescent DNA dye DRAQ5 (blue) was used to visualize nuclei. The scale bars represent 20 µm.* ***A****. Original magnification 40X (scale bar,).* ***B****. Higher magnification of selected area.*

2. Cosinor analysis of P-gp mRNA and protein expression datasets

ILEUM	**Mesor** (mRNA/36B4 for mRNAs, arbitrary units for proteins)	**Harmonics (h)**	**p**	**Amplitude** (mRNA/36B4 for mRNAs, arbitrary units for proteins)	**Phase (h min)**
**F, Abcb1a**	3.14 [2.43, 3.84]	24	0.0017	1.95 [0.95, 2.95]	11h38 [9h35, 13.42]
		12	0.09		
		8	0.23		
**M, Abcb1a**	2.37 [1.89, 2.85]	24	0.042	0.87 [0.18, 1,55]	13h17 [9h52, 16h43]
		12	0.71		
		8	0.86		
**F, Abcb1b**	5.09 [4.09, 6.09]	24	<0.001	3.43 [2.03, 4.83]	12h47 [11h10, 14h24]
		12	0.22		
		8	0.69		
**M, Abcb1b**	5.27 [4.05, 6.48]	24	0.02	2.46 [0.68, 4.24]	14h01 [11h4, 16h58]
		12	0.97		
		8	0.82		
**F, P-gp**	115.47 [107.45, 123.48]	24	<0.001	36.99 [25.64, 48.35]	14h44 [13h32, 15h55]
		12	<0.001	31.06 [19.54, 42.58]	3h43 [3h, 4h25]
		8	<0.001	24.87 [13.52, 36.22]	7h04 [6h27, 7h40]
**M, P-gp**	88.195 [82.38, 94.01]	24	0.14		
		12	0.002	14.79 [6.63, 22.93]	5h38 [4h31, 6h46]
		8	0.001	15 [7.17, 22.83]	1h40 [0h55, 2h25]

Table S1: **Cosinor analysis of P-gp mRNA and protein levels in the ileum of male and female mice**

COLON	**Mesor** (mRNA/36B4 for mRNAs, arbitrary units for proteins)	**Harmonics (h)**	**p**	**Amplitude** (mRNA/36B4 for mRNAs, arbitrary units for proteins)	**Phase (h min)**
**F, Abcb1a**	1.09 [0.64, 1,53]	24	0.33		
		12	0.98		
		8	0.98		
**M, Abcb1a**	2.66 [2.1, 3.22]	24	0.38		
		12	0.51		
		8	0.83		
**F, Abcb1b**	2.88 [1.04, 3.72]	24	0.19		
		12	0.86		
		8	0.76		
**M, Abcb1b**	8.05 [5.83, 10.28]	24	0.35		
		12	0.69		
		8	0.87		
**F, P-gp**	71.13 [66.06, 76.2]	24	0.14		
		12	<0.001	15.81 [8.86, 22.75]	3h07 [2h13, 4h02]
		8	0.93		
**M, P-gp**	66.45 [61.29, 71.61]	24	0.73		
		12	0.56		
		8	0.76		

Table S2: **Cosinor analysis of P-gp mRNA and protein levels in the colon of male and female mice**

LIVER	Mesor (mRNA/36B4 for mRNAs, arbitrary units for proteins)	Harmonics (h)	p	Amplitude (mRNA/36B4 for mRNAs, arbitrary units for proteins)	Phase (h min)
**F, P-gp**	2.89 [2.35, 3.42]	24	0.023	1.07 [0.34, 1.81]	0h25 [-2h35, 3h24]
		12	<0.001	1.68 [0.95, 2.40]	4h19 [3h24, 5h14]
		8	0.04	1.01 [0.24, 1.77]	3h35 [2h29, 4h40]
**M, P-gp**	3.27 [2.68, 4,05]	24	0.23		
		12	0.16		
		8	0.32		

Table S3: **Cosinor analysis of P-gp hepatic protein levels in male and female mice**

3. Talinolol PK parameters

The Area Under the Curve (AUC) over the first 8 h and the maximum concentration Cmax of Talinolol PK curves were computed for each mouse category (male/female, fed/fasted, ZT3/ZT15, plasma/liver/ileum). Briefly, virtual talinolol time-concentration profiles were generated by selecting one individual PK measurement for each time point and combining them for all timepoints. This was done for all possible combinations and yielded approximately 5000 virtual curves from which the AUC and Cmax values were computed. Mean and standard deviations of these values were then computed (Table S4).

		MALE				FEMALE			
		Fed		Fasted		Fed		Fasted	
		ZT3	ZT15	ZT3	ZT15	ZT3	ZT15	ZT3	ZT15
**plasma**	**AUC mean (µM.h)**	0.03	0.0255	0.0316	0.0223	0.0228	0.0165	0.0318	0.0266
	**AUC SD (µM.h)**	0.0039	0.004	0.0073	0.0047	0.0036	0.0039	0.0046	0.0062
	**Cmax mean (µM)**	0.0179	0.0145	0.0165	0.0168	0.0103	0.0092	0.0153	0.0153
	**Cmax SD (µM)**	0.0039	0.0025	0.0049	0.0035	0.0028	0.0017	0.0034	0.0039
**liver**	**AUC mean (µM.h)**	1.4535	1.4227	1.3301	1.4204	0.9886	0.7882	1.1544	0.8484
	**AUC SD (µM.h)**	0.2721	0.1867	0.2046	0.2965	0.2787	0.2028	0.2122	0.217
	**Cmax mean (µM)**	0.8574	0.8844	0.8052	1.3485	0.4577	0.4814	0.569	0.4788
	**Cmax SD (µM)**	0.2808	0.1092	0.2078	0.2555	0.192	0.1588	0.1404	0.1078
**ileum**	**AUC mean (µM.h)**	34.185	18.4591	29.8025	16.4763	13.541	7.9622	16.0401	9.989
	**AUC SD (µM.h)**	9.2781	6.2834	7.5191	5.3921	4.4424	2.1401	3.415	3.3413
	**Cmax mean (µM)**	19.8318	13.8113	14.9647	13.1515	7.3344	5.062	8.1167	5.6127
	**Cmax SD (µM)**	6.7568	4.5081	2.4153	7.629	2.0968	1.6356	2.6303	1.7817

Table S4: **AUC and Cmax values of Talinolo PK curves.** SD= Standard deviation.

4. Mechanistic Model of talinolol pharmacokinetics

A mathematical model of talinolol PK was designed at the whole-body level, and ultimately aimed to quantify P-gp activity in the gastro-intestinal system, where physiological P-gp expression is usually highest. Only talinolol was considered as the drug metabolites appear in negligible quantities in mice (11). The model simulates talinolol blood and biliary transport in between eight compartments that represent blood, liver, and duodenum, jejunum and ileum membranes and lumens (Fig. 5A). Talinolol oral administration was modeled as an input forcing function in the duodenum lumen representing the exponential emptying of the stomach (12). The drug efflux from the intestinal cells to the intestinal lumen was modeled as a *P-gp*-mediated active transport. Similarly, the entero-hepatic circulation from the liver to the intestinal lumen towards the biliary system was modeled as an active efflux involving liver *P-gp*. All other drug transports were modeled as passive diffusion through the tissues. In particular, the drug efflux from the liver towards blood was considered as passive as no experimental evidence exists regarding any interactions between talinolol and the main liver transporters involved in drug efflux towards the blood, including *Oat2*, *Oatp2b1*, *Mrp 1*, *3*, *4*, *5*, or *6*. All passive transport rates are modelled using Fick's first law whereas active transport follows Michaelis-Menten kinetics.

The model further considers talinolol renal and intestinal clearance that are represented as first-order kinetics in the blood and the ileum lumen compartments respectively. Although *P-gp* is expressed in the kidneys, talinolol renal excretion is assumed to be *P-gp* independent as i) the co-administration of verapamil, a *P-gp* inhibitor, did not modify talinolol renal clearance in humans and ii) talinolol renal elimination was highly correlated to the urinary clearance of creatinine, an amino-acid that is eliminated through passive diffusion from blood to primary urine in both healthy volunteers and in patients with renal failure (13-15).

We here describe the equations of the physiologically-based model of talinolol whole body PK. T_X denotes talinolol concentrations in the organ X, expressed in µM. The model equations are as follows:

$$V_{blood}\frac{\mathrm{d\,T_{blood}}}{\mathrm{dt}} = -(3 * \mathrm{k_{inGut}} + \mathrm{k_{inLiver}})\mathrm{T_{blood}} - \mathrm{k_{clear1}}(t)\mathrm{T_{blood}} + \mathrm{k_{outLiver}}\mathrm{T_{liver}}$$

$$V_{liver}\frac{\mathrm{d\,T_{liver}}}{\mathrm{dt}} = \mathrm{k_{inLiver}}\mathrm{T_{blood}} - \mathrm{k_{outLiver}}\mathrm{T_{liver}} + \mathrm{k_{portal}}(\mathrm{T_{duodenum}} + \mathrm{T_{jejunum}} + \mathrm{T_{ileum}}) - \frac{\mathrm{k}_{hepaticPgp}(t)\mathrm{T_{liver}}}{K_{hepaticPgp} + \mathrm{T_{liver}}}$$

$$V_{stomach}\frac{\mathrm{d\,T_{stomach}}}{\mathrm{dt}} = -\lambda(t)\mathrm{T_{stomach}}$$

$$V_{duodenum}\frac{\mathrm{d\,T_{duodenum}}}{\mathrm{dt}} = \mathrm{k_{inGut}}\,\mathrm{T_{blood}} + \mathrm{k_{lumen}}\mathrm{T}_{duoLumen} - \mathrm{k_{portal}}\,\mathrm{T_{duodenum}} - \frac{\mathrm{k}_{intestinPgp}(t)\mathrm{T_{duodenum}}}{K_{intestinPgp} + \mathrm{T_{duodenum}}}$$

$$V_{duoLumen}\frac{\mathrm{d\,T_{duoLumen}}}{\mathrm{dt}} = \lambda(t)(\mathrm{T_{stomach}} - \mathrm{T_{duoLumen}}) - \mathrm{k_{lumen}}\mathrm{T_{duoLumen}} + -\frac{\mathrm{k}_{intestinPgp}(t)\mathrm{T_{duodenum}}}{K_{intestinPgp} + \mathrm{T_{duodenum}}} + \frac{\mathrm{k}_{hepaticPgp}(t)\mathrm{T_{liver}}}{K_{hepaticPgp} + \mathrm{T_{liver}}}$$

$$V_{jejunum}\frac{\mathrm{d\,T_{jejunum}}}{\mathrm{dt}} = \mathrm{k_{inGut}}\,\mathrm{T_{blood}} + \mathrm{k_{lumen}}\mathrm{T}_{jejLumen} - \mathrm{k_{portal}}\,\mathrm{T_{jejunum}} - \frac{\mathrm{k}_{intestinPgp}(t)\mathrm{T_{jejunum}}}{K_{intestinPgp} + \mathrm{T_{jejunum}}}$$

$$V_{jejLumen}\frac{\mathrm{d\,T_{jejLumen}}}{\mathrm{dt}} = \lambda(t)(\mathrm{T_{duoLumen}} - \mathrm{T_{jejLumen}}) - \mathrm{k_{lumen}}\mathrm{T_{jejLumen}} + -\frac{\mathrm{k}_{intestinPgp}(t)\mathrm{T_{jejunum}}}{K_{intestinPgp} + \mathrm{T_{jejunum}}}$$

$$V_{ileum}\frac{\mathrm{d\,T_{ileum}}}{\mathrm{dt}} = \mathrm{k_{inGut}}\,\mathrm{T_{blood}} + \mathrm{k_{lumen}}\mathrm{T}_{ileumLumen} - \mathrm{k_{portal}}\,\mathrm{T_{ileum}} - \frac{\mathrm{k}_{intestinPgp}(t)\,\mathrm{T_{ileum}}}{K_{intestinPgp} + \mathrm{T_{ileum}}}$$

$$V_{ileumLumen}\frac{\mathrm{d\,T_{ileumLumen}}}{\mathrm{dt}} = \lambda(t)\mathrm{T_{jejLumen}} - k_{clear2}(t)\mathrm{T_{ileumLumen}} - \mathrm{k_{lumen}}\mathrm{T_{ileumLumen}} + -\frac{\mathrm{k}_{intestinPgp}(t)\mathrm{T_{ileum}}}{K_{intestinPgp} + \mathrm{T_{ileum}}}$$

The drug is administered into the stomach at an initial concentration C_0, here 100mg/kg. The exponential parameter λ(t) represents the rate of stomach emptying. For the sake of simplicity, it was set equal to the rate of drug progression along the duodenum, jejunum and ileum. Overall, λ(t) represents the drug progression from esophagus -where it is experimentally administered- along through the intestine.

Circadian rhythms (terms in blue) are assumed in *P-gp* activities in the intestine (efflux towards the intestinal lumen) and in the liver (efflux towards the bile) in agreement with experimental results of this study and literature. Thus, *P-gp* intracellular concentrations, in the three intestinal membrane compartments and in the liver, are represented by a cosine function considering the main period 24h and the two first harmonics at 12h and 8h. In addition, 24-h rhythms of the renal clearance were also implemented to account for daily variations in renal excretion (16, 17). Similarly, the drug stomach emptying, progression along the intestinal lumen compartments and intestinal clearance are assumed to vary over the 24h span with the same circadian amplitude and phase to account for daily variations in the gastro-intestinal tract motility (18, 19). Hence, in total, four circadian rhythms are considered in the model. Circadian rhythms in P-gp activity in the intestine and in the liver were modelled as:

$$k_{hepaticPgp}(t) = M_{hepaticPgp} + A_{hepaticPgp}^{24}\cos\left(\frac{2\pi}{24}\left(t - \varphi_{hepaticPgp}^{24}\right)\right)$$
$$+A_{hepaticPgp}^{12}\cos\left(\frac{2\pi}{12}\left(t - \varphi_{hepaticPgp}^{12}\right)\right)$$
$$+A_{hepaticPgp}^{8}\cos\left(\frac{2\pi}{8}\left(t - \varphi_{hepaticPgp}^{8}\right)\right)$$

$$k_{intestinPgp}(t) = M_{intPgp} + A_{intPgp}^{24}\cos\left(\frac{2\pi}{24}\left(t - \varphi_{intPgp}^{24}\right)\right)$$
$$+A_{intPgp}^{12}\cos\left(\frac{2\pi}{12}\left(t - \varphi_{intPgp}^{12}\right)\right)$$
$$+A_{intPgp}^{8}\cos\left(\frac{2\pi}{8}\left(t - \varphi_{ileumPgp}^{8}\right)\right)$$

24h-rhythms were also assumed in the renal clearance (k_{clear1}), in the intestinal excretion (k_{clear2}) and in the drug progression rate along the gastrointestinal tract (λ). We assumed that the variations in both k_{clear2} and λ resulted from circadian rhythms in gastrointestinal tract motility and thus had the same amplitude and phase. Equations are as follows:

$$k_{clear1}(t) = M_{clear1} + A_{clear1}\cos\left(\frac{2\pi}{24}\left(t - \varphi_{clear1}\right)\right)$$

$$k_{clear2}(t) = M_{clear2} + A_{clear2}\cos\left(\frac{2\pi}{24}\left(t - \varphi_{clear2}\right)\right)$$

$$\lambda(t) = M_{\lambda} + A_{clear2}\cos\left(\frac{2\pi}{24}\left(t - \varphi_{clear2}\right)\right)$$

5. Model parameters: organ volumes

Average male and female mouse weight were set as 0.025 kg and 0.023 kg respectively. Relative blood volume was assumed to be 58.5 mL/kg (20). The volume of the liver was set to 1.39 mL for male mice and 1.12 mL for female mice, irrespective of feeding conditions (21). Stomach volume was set to 0.37 mL for female mice (22) and at a 17% greater value for male mice. Duodenum volume was estimated as 0.2295 mL for male mice and 0.1905 mL for females (23). Jejunum volumes were set to 0.7541 mL for males and 0.6259 mL for females. Ileum volumes were set to 0.0765 mL for males and 0.0635mL for females. The ileum mucosa volume was computed according to information from literature as follows (24). The ileum total radius in mouse was approximated to $r_{total\_ileum} = 3500\mu m$ and the wall thickness to $r_{mucosa} = 200\mu m$. The ileum was assumed to have a cylinder shape so that the volume fraction corresponding to the ileum lumen was estimated by:

$$\frac{V_{ileum\_lumen}}{V_{total\_ileum}} = \frac{\pi(r_{total_{ileum}} - r_{mucosa})^2 l}{\pi r_{total\_ileum}{}^2 l} = \frac{(r_{total_{ileum}} - r_{mucosa})^2}{r_{total\_ileum}{}^2} = 0.8889 \approx 0.9$$

where *l* is the ileum length. Hence, the volume of the lumen was set to 90% of the total ileum volume and that of the mucosa to the remaining 10%. The same fractions were kept for the duodenum and jejunum compartments.

6. Model Results: estimated parameters for passive talinolol transport

The physiologically based model of talinolol chronoPK was used to recapitulate both ileum and hepatic P-gp protein and talinolol PK datasets towards analysis of P-gp activity at the whole organism level. A set of model parameters was estimated for each mouse class, namely male/female, fed/fasted animals. This allowed to conclude on variations according to sex, feeding conditions and circadian timing of all model parameters (Fig. 5, S3). Talinolol stomach emptying and progression along the intestine were faster in male mice compared to females which is in agreement with human data (Fig. S3A, (26, 27)). As for ileum P-gp activity circadian mean, passive absorption rate from ileum lumen to mucosa cells was higher in males compared to females (Fig. S3B). In both sexes, fasting decreased talinolol transport rates from liver to blood and from blood to ileum (Fig. S3D and F).

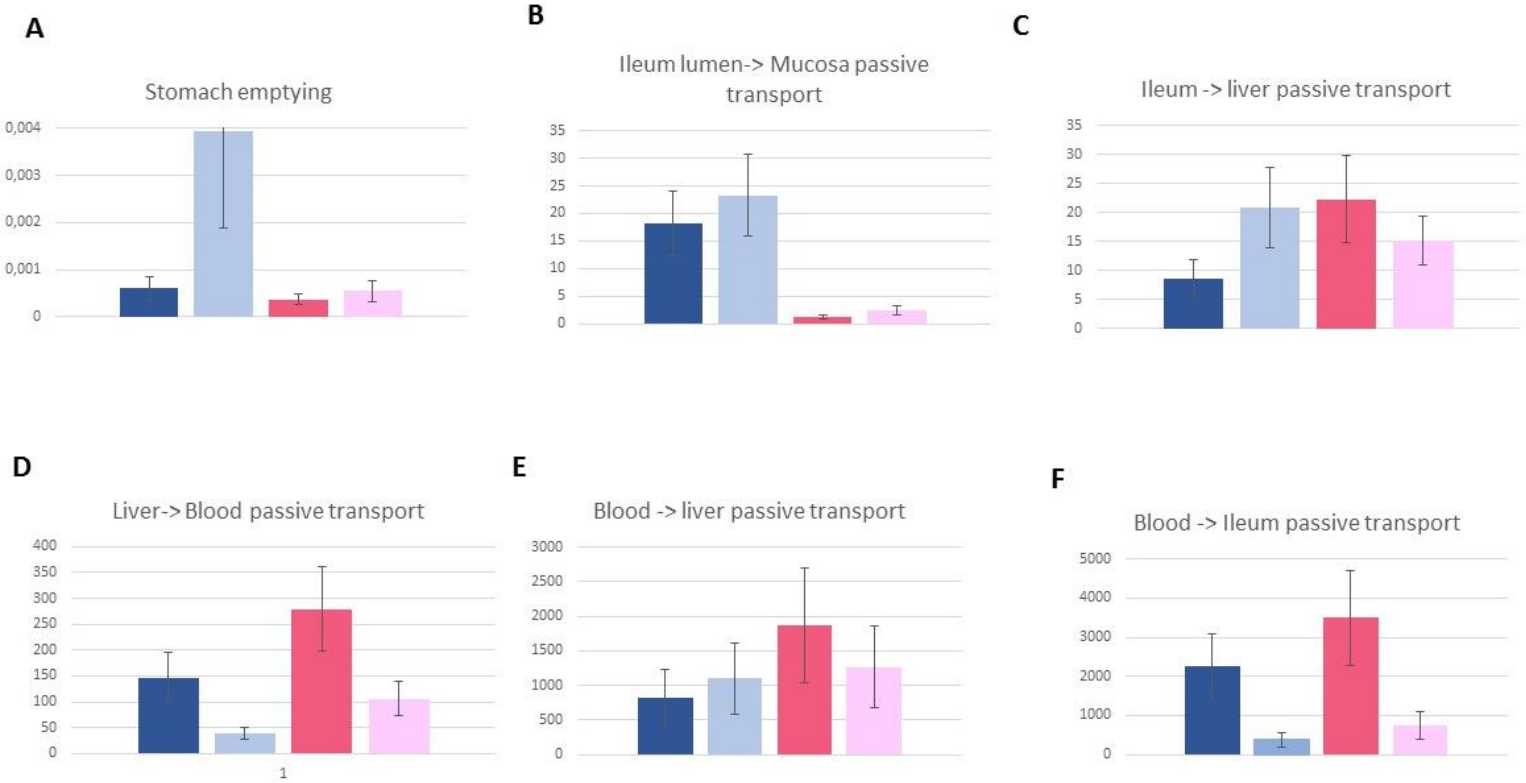

Figure S3. Talinolol chronoPK model parameters

References

1. Gu L, *et al.* (2009) A new model for studying tissue-specific mdr1a gene expression in vivo by live imaging. *Proceedings of the National Academy of Sciences of the United States of America* 106(13):5394-5399.
2. Chomczynski P & Sacchi N (2006) The single-step method of RNA isolation by acid guanidinium thiocyanate-phenol-chloroform extraction: twenty-something years on. *Nature protocols* 1(2):581-585.
3. Kononen J, *et al.* (1998) Tissue microarrays for high-throughput molecular profiling of tumor specimens. *Nat Med* 4(7):844-847.
4. Ando H, *et al.* (2005) Daily rhythms of P-glycoprotein expression in mice. *Chronobiology international* 22(4):655-665.
5. Murakami Y, Higashi Y, Matsunaga N, Koyanagi S, & Ohdo S (2008) Circadian clock-controlled intestinal expression of the multidrug-resistance gene mdr1a in mice. *Gastroenterology* 135(5):1636-1644.e1633.
6. Okyar A, *et al.* (2012) Circadian variations in exsorptive transport: in situ intestinal perfusion data and in vivo relevance. *Chronobiology international* 29(4):443-453.
7. Pathak SM, Musmade PB, Bhat KM, & Udupa N (2010) Validated HPLC method for quantitative determination of talinolol in rat plasma and application to a preclinical pharmacokinetic study. *Bioanalysis* 2(1):95-104.
8. Cornelissen G (2014) Cosinor-based rhythmometry. *Theoretical biology & medical modelling* 11:16.
9. Miller FP, Vandome AF, & McBrewster J (2010) *Nyquist-Shannon Sampling Theorem: Aliasing, Sine Wave, Signal Processing, Nyquist Rate, Nyquist Frequency, Sampling*

Rate, Shannon-Hartley Theorem, Whittaker-Shannon Interpolation Formula, Reconstruction from Zero Crossings (Alphascript Publishing).
10. Ballesta A*, et al.* (2011) A combined experimental and mathematical approach for molecular-based optimization of irinotecan circadian delivery. *PLoS Comput Biol* 7(9):e1002143.
11. Schupke H, Hempel R, Eckardt R, & Kronbach T (1996) Identification of talinolol metabolites in urine of man, dog, rat and mouse after oral administration by high-performance liquid chromatography-thermospray tandem mass spectrometry. *J Mass Spectrom* 31(12):1371-1381.
12. Schwarz R, Kaspar A, Seelig J, & Kunnecke B (2002) Gastrointestinal transit times in mice and humans measured with 27Al and 19F nuclear magnetic resonance. *Magn Reson Med* 48(2):255-261.
13. Staud F, Ceckova M, Micuda S, & Pavek P (2010) Expression and function of p-glycoprotein in normal tissues: effect on pharmacokinetics. *Methods Mol Biol* 596:199-222.
14. Krueger M*, et al.* (2001) Pharmacokinetics of oral talinolol following a single dose and during steady state in patients with chronic renal failure and healthy volunteers. *Int J Clin Pharmacol Ther* 39(2):61-66.
15. Schwarz UI*, et al.* (1999) Unexpected effect of verapamil on oral bioavailability of the beta-blocker talinolol in humans. *Clin Pharmacol Ther* 65(3):283-290.
16. Pons M, Tranchot J, L'Azou B, & Cambar J (1994) Circadian rhythms of renal hemodynamics in unanesthetized, unrestrained rats. *Chronobiology international* 11(5):301-308.
17. Noh JY*, et al.* (2011) Circadian rhythms in urinary functions: possible roles of circadian clocks? *International neurourology journal* 15(2):64-73.
18. Gschossmann JM*, et al.* (2001) Diurnal variation of abdominal motor responses to colorectal distension and plasma cortisol levels in rats. *Neurogastroenterology and motility : the official journal of the European Gastrointestinal Motility Society* 13(6):585-589.
19. Hoogerwerf WA*, et al.* (2010) Rhythmic changes in colonic motility are regulated by period genes. *Am J Physiol Gastrointest Liver Physiol* 298(2):G143-150.
20. Anonymous (2007) Small Laboratory Animals. *Handbook of Laboratory Animal Management and Welfare*, (Blackwell Publishing Ltd), pp 233-271.
21. Marino DJ (2012) Age-specific absolute and relative organ weight distributions for B6C3F1 mice. *J Toxicol Environ Health A* 75(2):76-99.
22. McConnell EL, Basit AW, & Murdan S (2008) Measurements of rat and mouse gastrointestinal pH, fluid and lymphoid tissue, and implications for in-vivo experiments. *J Pharm Pharmacol* 60(1):63-70.
23. Casteleyn C, Rekecki A, Van der Aa A, Simoens P, & Van den Broeck W (2010) Surface area assessment of the murine intestinal tract as a prerequisite for oral dose translation from mouse to man. *Lab Anim* 44(3):176-183.
24. Navarrete J, Vásquez B, & del Sol M (2015) Morphoquantitative analysis of the Ileum of C57BL/6 mice (Mus musculus) fed with a high-fat diet. *International Journal of Clinical and Experimental Pathology* 8(11):14649-14657.
25. Dulong S, Ballesta A, Okyar A, & Levi F (2015) Identification of Circadian Determinants of Cancer Chronotherapy through In Vitro Chronopharmacology and Mathematical Modeling. *Mol Cancer Ther*.
26. Bebawy M & Chetty M (2009) Gender differences in p-glycoprotein expression and function: effects on drug disposition and outcome. *Current drug metabolism* 10(4):322-328.

27. Degen LP & Phillips SF (1996) Variability of gastrointestinal transit in healthy women and men. *Gut* 39(2):299-305.
28. Worsøe J, *et al.* (2011) Gastric transit and small intestinal transit time and motility assessed by a magnet tracking system. *BMC Gastroenterology* 11(1):145.
